# Computational Study into the Effects of Countercations on the [P_8_W_48_O_184_]^40–^ Polyoxometalate Wheel

**DOI:** 10.1021/acsorginorgau.3c00014

**Published:** 2023-07-22

**Authors:** Daniel Malcolm, Laia Vilà-Nadal

**Affiliations:** † School of Chemistry, University of Glasgow, Glasgow G12 8QQ, United Kingdom

**Keywords:** cluster chemistry, molecular structure, electronic structure, computational chemistry, polyoxometalate benchmarking

## Abstract

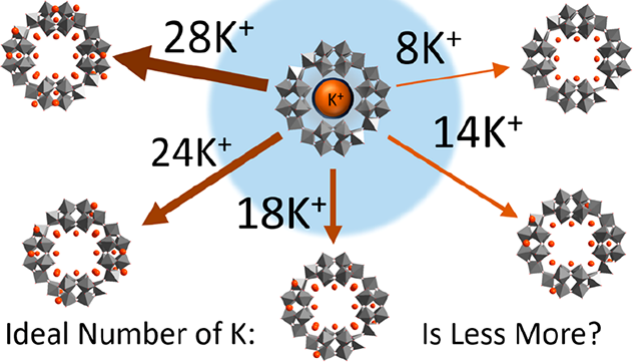

Porous metal oxide materials have been obtained from a ring-shaped macrocyclic polyoxometalate (POM) structural building unit, [P_8_W_48_O_184_]^40–^. This is a tungsten oxide building block with an integrated “pore” of 1 nm in diameter, which, when connected with transition metal linkers, can assemble frameworks across a range of dimensions and which are generally referred to as POMzites. Our investigation proposes to gain a better understanding into the basic chemistry of this POM, specifically local electron densities and locations of countercations within and without the aforementioned pore. Through a rigorous benchmarking process, we discovered that 8 potassium cations, located within the pore, provided us with the most accurate model in terms of mimicking empirical properties to a sufficient degree of accuracy while also requiring a relatively small number of computer cores and hours to successfully complete a calculation. Additionally, we analyzed two other similar POMs from the literature, [As_8_W_48_O_184_]^40–^ and [Se_8_W_48_O_176_]^32–^, in the hopes of determining whether they could be similarly incorporated into a POMzite network; given their close semblance in terms of local electron densities and interaction with potassium cations, we judge these POMs to be theoretically suitable as POMzite building blocks. Finally, we experimented with substituting different cations into the [P_8_W_48_O_184_]^40–^ pore to observe the effect on pore dimensions and overall reactivity; we observed that the monocationic structures, particularly the Li_8_[P_8_W_48_O_184_]^32–^ framework, yielded the least polarized structures. This correlates with the literature, validating our methodology for determining general POM characteristics and properties moving forward.

## Introduction

Molecular metal oxides, commonly referred to as polyoxometalates (POMs), are ordered, inorganic metal-oxide clusters,^1,2^ with the general formula [MO_*x*_]_*n*_ (where M = Mo, W, V, Nb, *x* = 3–7, *n* = 6–368).^3^ These clusters spontaneously self-assemble from oxometalate monomers under appropriate, usually acidic, reaction conditions.^4^ The range of potential applications for POMs spans many different fields and disciplines including use within biological systems,^5,6^ as components within supramolecular assemblies,^7,8^ as catalysts,^9,10^ as electron storage molecules,^11,12^ and for increasing the storage capabilities of portable memory devices.^13^

**Figure 1:**
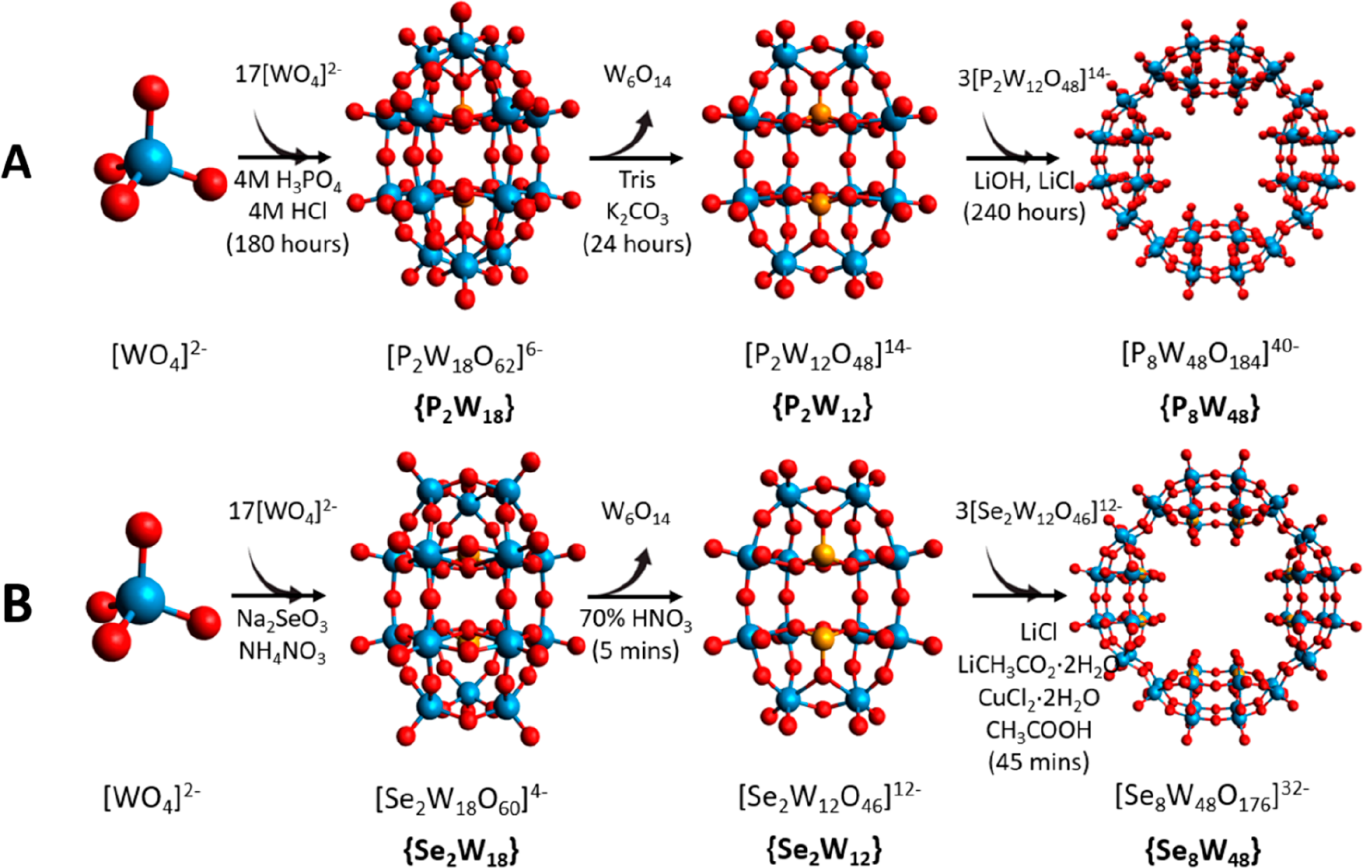
Synthesis of (A) [P_8_W_48_O_184_]^40–^ via [P_2_W_18_O_62_]^6–^ WD and [P_2_W_12_O_48_]^14–^ lacunary intermediates^16,17^ and (B) [Se_8_W_48_O_176_]^32–^ via [Se_2_W_18_O_60_]^4–^ WD and [Se_2_W_12_O_46_]^12–^ lacunary intermediates.^23^ Basic conditions exist only during step 2, during which the framework loses roughly one-fourth of its structure.

One particularly promising area of application is porosity/adsorption, where the wheel-shaped POM [P_8_W_48_O_184_]^40–^, shortened to {P_8_W_48_},^14^ is an especially good candidate owing to its relatively large central pore (1 Å/0.1 nm diameter), exceptional electrochemical properties, unusually high anionic charge, and robust synthetic route (Figure 1).^14−16^ The central pore can be utilized for several different purposes, including encapsulating countercations^17^ and providing a scaffold for a multimetal construction, where the metal is Fe,^18^ or Cu,^19,20^ that confers magnetic properties to the POM as a whole. A {P_8_W_48_} wheel is formed from 4 [P_2_W_12_O_48_]^14–^ hexa-vacant lacunary units, shortened to {P_2_W_12_}, each of which originates from a Wells-Dawson (WD) type framework (P_2_W_18_O_62_]^6–^), abbreviated as {P_2_W_18_},^16,17^ after it has been exposed to mildly basic conditions. WD species are frequently utilized in POM chemistry due to their relative stability, which extends across 6 isomers and a large combination of heteroatoms and lacunary sites.^21,22^ Other than the {P_2_W_18_} WD already outlined, 2 other WDs capable of forming stable hexalacunaries after basic degradation have been identified: [Se_2_W_18_O_60_]^4–^^23^ and [As_2_W_18_O_62_]^6–^.^24^ Creation of these lacunary sites under basic conditions allows for the insertion of new transition metal elements not currently present in the POM, enabling the capability for fine-tuning of the redox properties, catalytic localization and activation of the framework,^22^ and assembly of the POM into a building block suitable for POMzite construction.^23,25,26^

In 2017, Boyd et al. demonstrated how {P_8_W_48_} POMs can additionally be used to construct a species of porous nanomaterial commonly referred to as a POMzite.^26^ In these POMzites, individual POMs are linked together by transition metal (TM) oxide anions of the form [MO_*x*_]^q–^^27^ (where M = Mn,^28,29^ Co,^27^ V,^27^ Ni,^26^ Ag,^30^*x* = 4–6). The pairing of a rigid structure with a high degree of customizability grants POMzites the capacity to rival similar porous materials, such as zeolites and metal organic frameworks (MOFs);^31^ displaying the benefits of both while suffering from the disadvantages of neither.^32^

One particularly promising study by Zhan et al. illustrates this point perfectly, where the POMzite synthesized was capable of existing as one of 8 distinct states and of transforming from one state to another when exposed to changes in NH_3_ or CH_3_OH concentration or in temperature/hydration.^33^ Each conformation has different absorption properties and stabilities, allowing for precise tuning and enhancement of functionality for a particular application. This inherent ability of a material to repeatedly alter its crystallinity and properties based on controllable stimuli has the potential to revolutionize the materials industry.

Compared with zeolites, the elements that compose POMzites are rarer,^34^ which, combined with the lack of a comprehensive understanding of their self-assembly processes, acts to drive up the costs associated with researching these materials. Tackling this problem in a brute force manner in the lab promises to therefore be a costly endeavor,^35^ with no guarantee of yielding an optimal POMzite capable of recuperating financial losses. Consequently, an alternate method is required that reduces the fiscal burden either by limiting synthetic costs^36^ or by employing a computational approach which reduces lab time.

Inverse design^37,38^ is a method that has the potential to provide a workaround; designing a molecule or material in this manner requires a theoretical model of the product, one that is expected to fulfill a specific role, followed by working backward along the synthetic route until the reagents and reaction conditions necessary to yield the desired product are elucidated. It also allows for easy experimentation with novel ideas, testing the waters to determine if an approach is feasible before expensive lab work is undertaken.^17,39^ This approach, however, requires a greater understanding of the self-assembly process in order to accurately simulate both real and hypothetical structures.^40^ If we are to properly apply the tenets of inverse design, we must have the capabilities to build and analyze complex computational models of POMs and have a deep knowledge of their self-assembly processes.^41^

Herein we present our {X_8_W_48_} wheel, {X_2_W_18_} WD and {X_2_W_12_} hexalacunary calculation-based data, complete with HOMO–LUMO gap energy values, molecular electrostatic potential maps (MEPs), and standard deviation (STD) calculations. This is intended to deepen our fundamental understanding of these molecules so as to simulate them and currently theoretical but chemically promising derivatives more accurately.

## Computational Details

All calculations were performed using the Amsterdam Modeling Suite (AMS 2023.1).^42,43^ In this work, we have been comparing the results from using several different functionals: the nonempirical Perdew–Burke–Ernzerhof (PBE), the generalized gradient approximation (GGA) exchange-correlation functional,^44^ the empirical exchange-correlation functionals of Becke and Perdew,^45,46^ the B3LYP hybrid functionals,^47^ the PBE0 functional of Adamo and Barone,^48,49^ a dispersion correction in the form of DFT-D2,^50^ which was applied to PBE (PBE-D) and B3LYP (B3LYP-D), the range separated functional wB97X,^51^ and the Minnesota 2006 local functional (M06-L).^52^ Relativistic corrections were included by means of the ZORA formalism.^53^ Triple-ζ polarization (def2- TZP),^54^ triple-ζ plus polarization (def2-TZ2P), and (def2-QZ4P) basis sets were employed to describe the valence electrons of all atoms, all of which were from the ADF basis set library.^55^ Structures were optimized in the presence of a continuous model solvent by means of the conductor-like screening model (COSMO), with water as a solvent.^56,57^

The solvation radii values used were the standard van der Waals values for ADF,^58^ determined by Alvarez.^59^ Computational results were obtained using the ARCHIE-WeSt High Performance Computer (see www.archie-west.ac.uk) based at the University of Strathclyde.

## Results and Discussion

Initially, we tested the accuracy and time effectiveness of the computational methods available in SCM-ADF.^41^ We chose our functionals and basis sets from a broad range of methods, some of which had been previously used in other computational POM studies. The most used functional in the literature was PBE,^41,60−62^ as established by Swart and co-workers in their 2021 review;^63^ in terms of basis sets, the literature indicated that def2-TZVP is the best choice, although TZP acts as a substitute due to def2-TZVP not being included in the ADF package.^23,64,65^ We also compared the effects of using either no, small, or large frozen cores and of single point vs optimization tasks.

To this end, we utilized GGA (PBE, BP86^41^), GGA-D (PBE-D), hybrid (B3LYP,^41^ B3LYP-D, PBE0^41,66^), and range separated functional(s) (wB97x), and a few basis sets (TZP,^62,66^ TZ2P,^66^ QZ4P^66^) to obtain a broad distribution of results and to aid in selecting the appropriate level of theory for our purposes.

**Figure 2:**
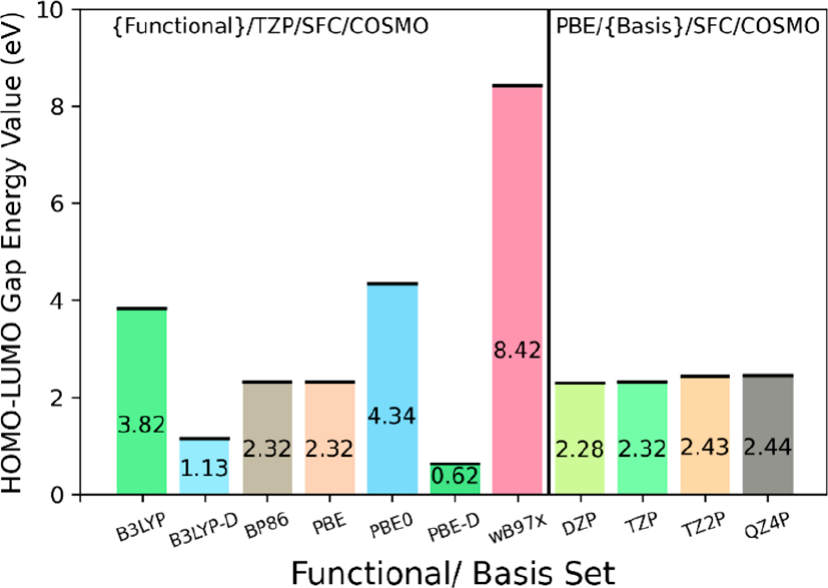
HOMO–LUMO gap energy values for a range of functionals and basis sets. The data presented here are for {P_2_W_18_}. Functional calculations were run with a TZP basis set, small frozen cores, and the COSMO model with water as a solvent; these conditions were maintained for the basis set calculations, with the only difference being PBE as the functional.

The functionals which most closely corresponded to the empirical data out of the range tested were PBE and BP86 (Figure 2). Going forward, we chose to use a GGA (PBE) functional and TZP basis set, paired with a small frozen core and a COSMO solvation model^67^ as part of an optimization task (see SI-1 for full results, as well as for details on fragments and results of benchmarking).

**Figure 3:**
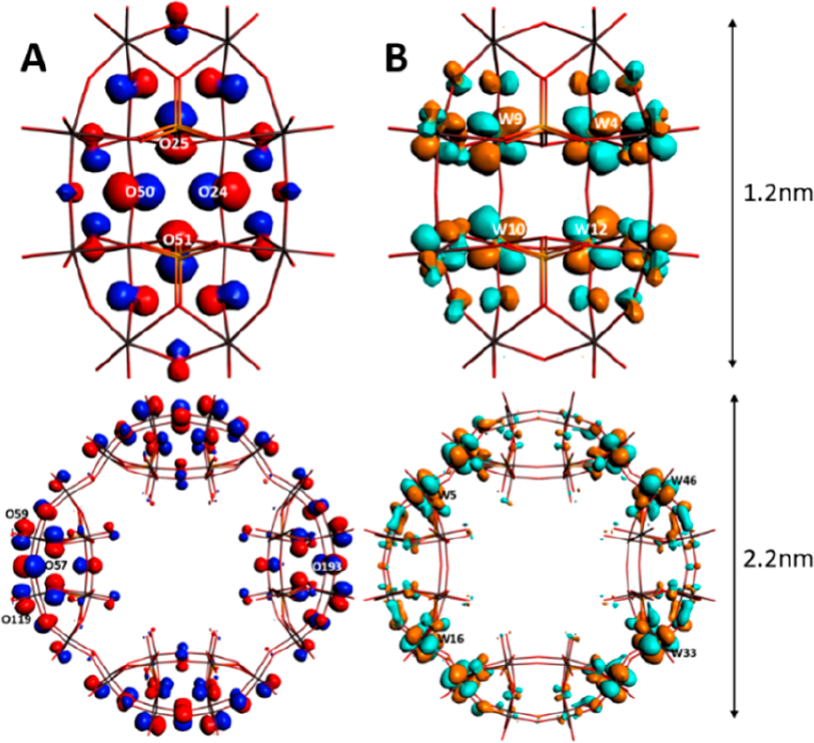
Visualization of HOMO and LUMO orbitals for {P_2_W_12_} (A) and {P_8_W_48_} (B), with labeling of the 4 atoms that contribute most to their respective orbital band.

**Figure 4:**
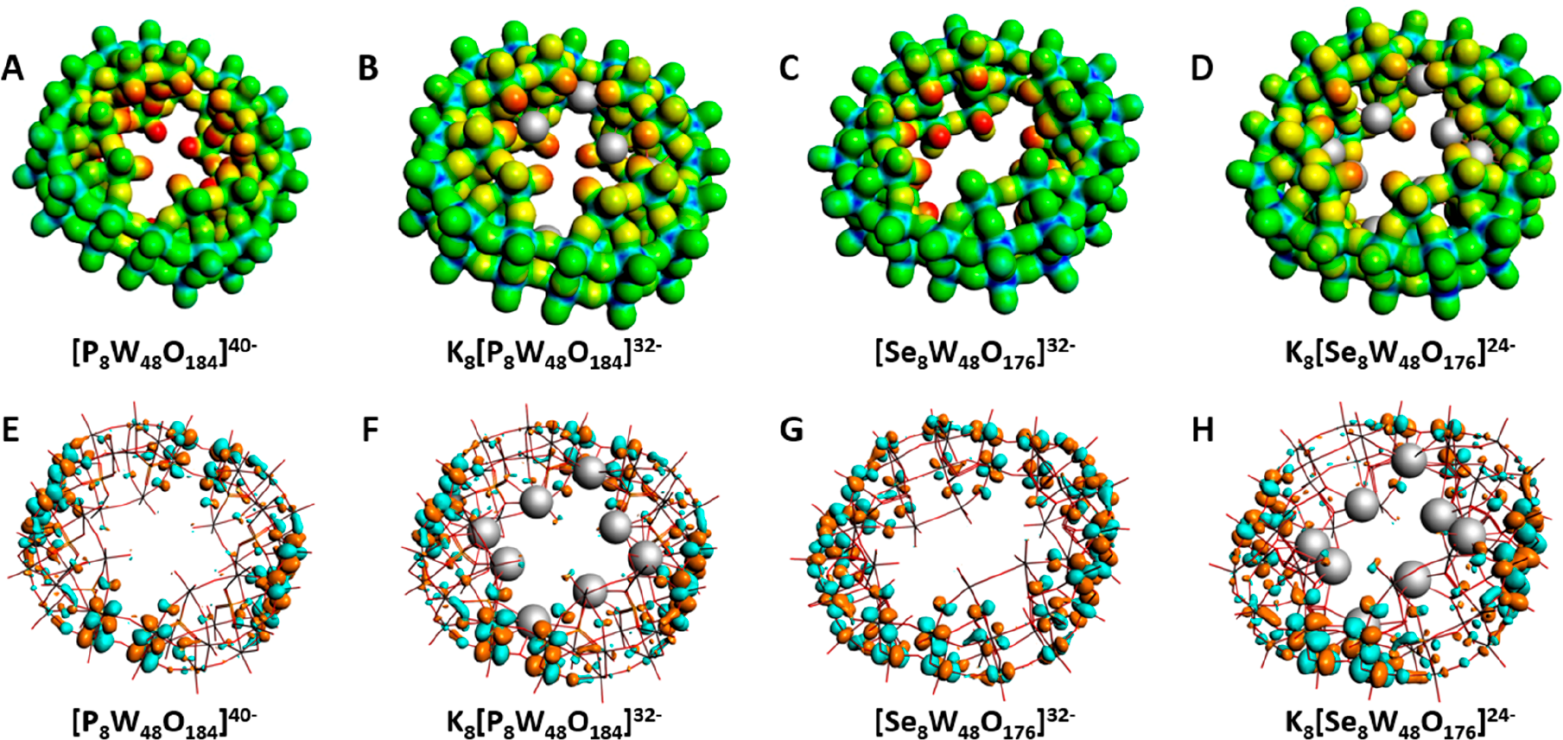
MEPs for [P_8_W_48_O_184_]^40–^ (A), K_8_[P_8_W_48_O_184_]^32–^ (B), [Se_8_W_48_O_176_]^32–^ (C), and K_8_[Se_8_W_48_O_176_]^24–^ (D) and LUMO visualizations for [P_8_W_48_O_184_]^40–^ (E), K_8_[P_8_W_48_O_184_]^32–^ (F), [Se_8_W_48_O_176_]^32–^ (G), and K_8_[Se_8_W_48_O_176_]^24–^ (H). Geometries were optimized and MEP generated with DFT/PBE/SFC/TZP. More nucleophilic (negative) regions are visualized in red, and more electrophilic (positive) regions are visualized in blue. The color ranges for the MEPs are −2.40 to −1.50 (A), −1.90 to −1.20 (B), −1.80 to −1.20 (C), and −1.40 to −0.73 (D). Basis sets, TZ2P and QZ4P, were observed to alter the HOMO–LUMO gap value only negligibly and took at least 9 times longer to converge.

To complete our benchmarking, we also compared the accuracy of our results against work done by Cameron et al.,^23^ Zhang et al.,^68^ and Vilà-Nadal and co-workers.^69,70^ Our results were very close in value to those of Vilà-Nadal and Zhang, and, despite a larger difference in values caused by different computational software, were in good agreement with the Cameron work as well. Our optimization calculations of {P_8_W_48_} and {P_2_W_12_} yielded the nature of the HOMO and LUMO orbitals (Figure 3), as well the energy gap between them,^71,72^ and the molecular electrostatic potentials (MEPs) (Figure 4). The HOMOs for both POMs receive their greatest collective atomic contribution from the framework oxygens, specifically the P_Y_ orbitals, located “behind” the heteroatoms in the center of the POM. This is a typical distribution observed in POMs, as described in an RSC paper by Poblet et al.^73^ In addition to {P_8_W_48_}, we also ran calculations on [Se_8_W_48_O_176_]^32–^ and [As_8_W_48_O_184_]^40–^, both of which have been previously reported in the literature (see SI-3 for {As_8_W_48_} and {Se_8_W_48_} HOMO and LUMO visualizations, and SI-4 and -7 for the electronic values for all hexalacunary and {P_8_W_48_} POMs featured in this work).^23,74,75^

In contrast, the position of the LUMO orbitals differs between the {P_2_W_12_} and {P_8_W_48_} structures; in the lacunary (Figure 3B), they are localized on the tungsten atoms, the D_YZ_ orbital specifically, connected to the main HOMO oxygens, forming a complete square at the back of the lacunary. With the full {P_8_W_48_} wheel, however, the LUMO shifts onto the tungsten atoms which link each lacunary quarter of the wheel together (Figure 4E). The fact that successive reductions will increase electron density in these areas is key to understanding how injection of electrons affects regional stability, as it may influence the tendency of the ring to disintegrate back into its lacunary substituents if reduced to a high enough degree. The threshold at which this disintegration will occur is currently unknown but appears to be high, allowing for at least 18 or 27 successive reductions of {P_2_W_18_} or {P_8_W_48_} respectively.^76^

The MEPs revealed that the interior of the ring is more nucleophilic than the exterior, which explains why this POM is exceptionally good at trapping cations within its central pore (Figure 4A and C). This polarization of electrons is likely due to the phosphorus atom, which is in the closest proximity to terminal oxygen atoms along the interior face.

Countercations, such as those present in K_8_[P_8_W_48_O_184_]^32–^, are introduced during synthesis to stabilize the highly anionic charge of the framework; they achieve this by situating themselves near the highly nucleophilic oxygen atoms, thereby reducing the high degree of polarization and increasing the overall electrophilicity of the POM. This is clearly visible in Figure 4B and D, where the “red hot” nucleophilic region in the center diffuses over more of the POM, visible as an orange section. A small reduction in nucleophilicity is visible for {Se_8_W_48_} around the “hinge” areas where the lacunaries link up compared to {P_8_W_48_}; this is due to {Se_8_W_48_} lacking 2 oxygens per hinge region in these areas. With K_8_{P_8_W_48_}, the presence of the countercations within the highly nucleophilic in the interior face is reduced in intensity, closer to that of the surrounding areas.

The MEPs we obtained were in good agreement with the literature, where cations/cationic scaffolds assemble within the pore specifically; the works of Ulrich Kortz,^20,77^ and Thomas Boyd,^17^ spring to mind. Thus, we can prove that we can produce an accurate DFT-level model of {P_8_W_48_}. LUMO visualizations in Figure 4E and F are almost identical, indicating that inclusion of countercations has a minimal effect on the position and intensity of the LUMO orbitals. There is, however, an observable, if slight, difference in LUMO localization between Figure 4E and F, implying that the choice of heteroatom can help tune reduction, specifically where it occurs (see SI-5, -6, and -8 for the full range of MEPs obtained during this work).

**Figure 5:**
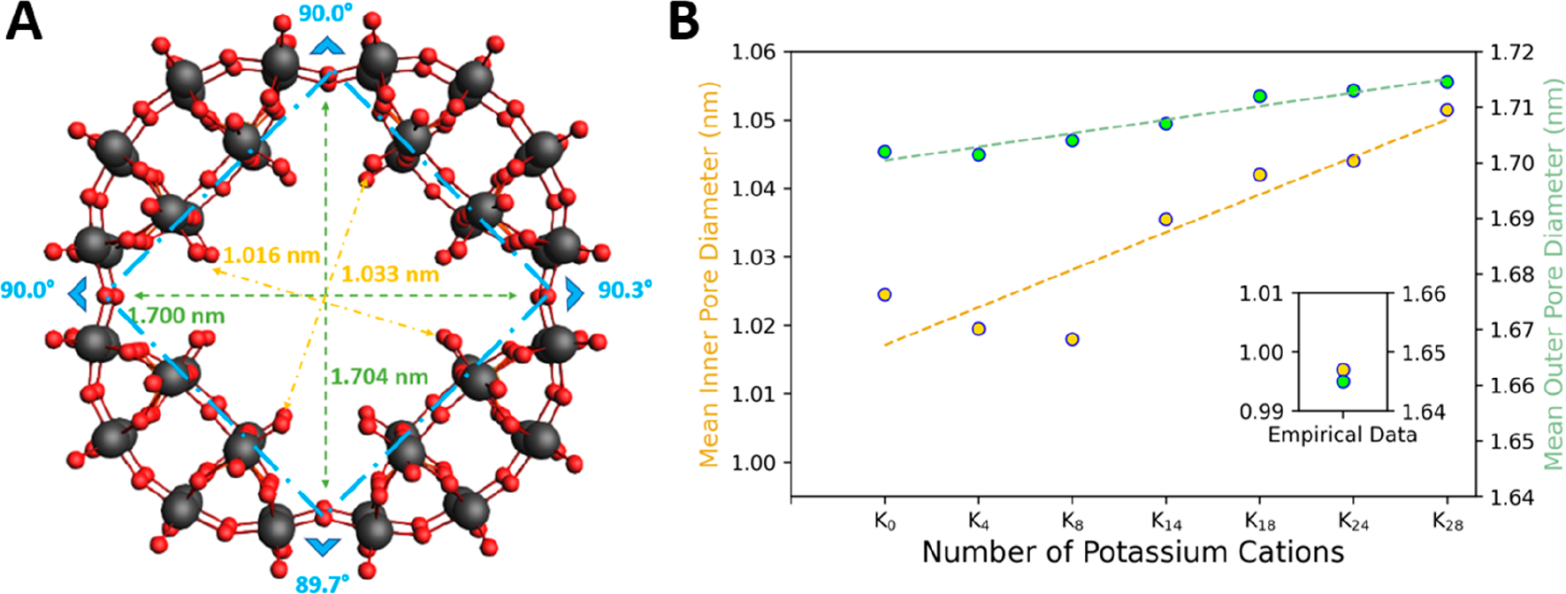
Measurement of angles and diameters within the [P_8_W_48_O_184_]^40–^ (A) wheel, with angles (blue), the smaller, “inner”, pore diameter (yellow), and the larger, “outer”, pore diameter (green). (B) The pore diameter increases as more cations are included in the {P_8_W_48_} structure. A subplot is included that shows the experimentally obtained values in relation to those measured from calculations.

We initially utilized only 8 K countercations in our geometry, yet the full formula for {P_8_W_48_} is K_28_Li_5_H_7_[P_8_W_48_O_184_]; the models we have constructed thus far may be less than accurate at describing the true structure, especially given that our benchmarking for {P_8_W_48_} has not been conducted against empirical data. To rectify this, we built several K_*n*_{P_8_W_48_} frameworks, progressively adding K countercations every time. We based the positions of each potassium cation on available POMzite xyz files,^26,78^ which can include up to 18 K cations per {P_8_W_48_} wheel, and added in the rest based on the principle of maintaining/improving symmetry of the overall structure (see Figures 5, S19, and S20). Symmetry is a key factor to keep in mind when designing a molecular model, as a more symmetrical system will take less effort to converge due to there being fewer unique interactions throughout the molecule to consider.

Determining the mean diameter of the {P_8_W_48_} from several points of experimental data, we compared this value against that from our optimized geometries (see SI-9 for the full list of structures used and the data extracted from them); we discovered that the structure with 8 countercations was the closest in diameter to the experimental mean. Inclusion or exclusion of more cations increased the diameter value. When also compared against the computational effort required to converge each geometry (Figure S20), it becomes clear that 8 K countercations yields the best reflection of reality while still being computationally efficient; an efficient calculation is able to reach the convergence point for a structure within a reasonable amount of time given the number of cores used. For molecules of a size akin to {P_8_W_48_}, a total calculation time of less than a week when 30 cores are used is considered efficient. Additionally, we compared our converged geometries for {As_8_W_48_} and {Se_8_W_48_} against the available empirical data, and both were in good agreement (see SI-10 and -11 for the full {As_8_W_48_} and {Se_8_W_48_} benchmarking data).

We theorize that the ideal model only contains 8 K cations due to the implicit limitations imposed by this computational modeling. Cations such as K are surrounded by a score of solvent molecules in solution, which prevents tight bonding occurring between the cation and POM; when using an effective solvation model such as COSMO, however, where the solvent is simulated via a medium, this aspect of solvent interaction is lost. The significance of this is that each cation in a theoretical model now exerts a much greater influence on the overall electron distribution and stability of the molecule than would be observed in solution.

**Figure 6:**
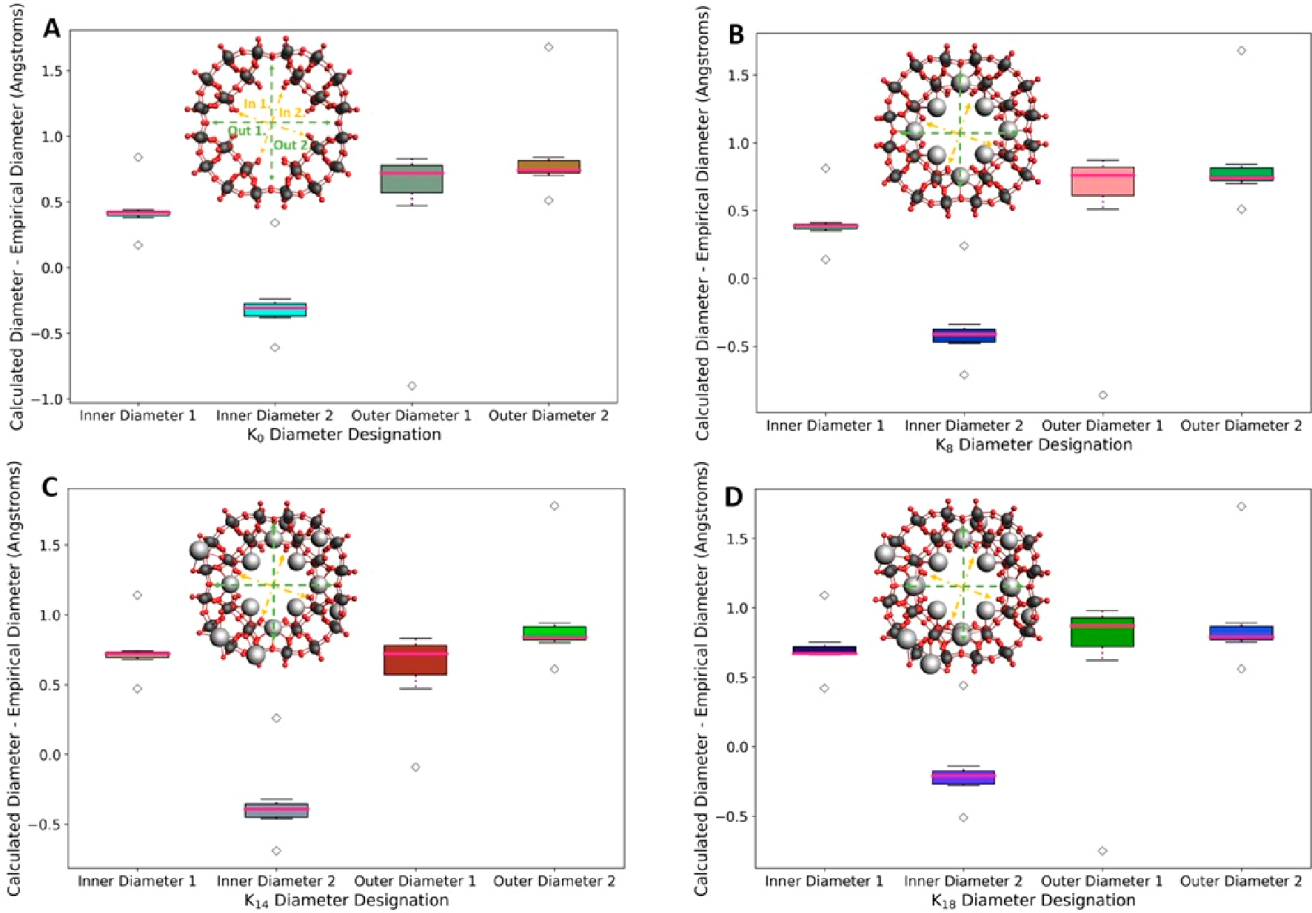
Boxplots showing difference between calculated and empirical diameters across a range of 4 distinct diameters for a [P_8_W_48_O_184_]^40–^ (A), K_8_[P_8_W_48_O_184_]^32–^ (B), K_14_[P_8_W_48_O_184_]^26–^ (C), and K_18_[P_8_W_48_O_184_]^22–^ (D) framework.

When the difference in diameter values between our theoretical models and the available empirical data is arranged into a boxplot (see Figure 6), we can observe several points of interest. First, there is little change in the proportions of the differences between calculated and empirical diameters as more cations are included in the structure; *inner diameter 2* and *outer diameters 1* and *2* barely change across the four boxplots. The exception is *inner diameter 1*, which experiences an increase in diameter between the addition of 8 and 14 potassium cations (see SI-12 for the full list of potassium countercation data, including the error values).

We theorize that this increase in the value of *inner diameter 1* is caused by the uneven addition of cations to the exterior of the POM; two hexalacunary sections receive 2 K cations, while the other two only benefit from one. This configuration results in a pinched geometry, with the two sections featuring 2 cations repelling more strongly from the similarly cationic pore than the other two.

**Figure 7:**
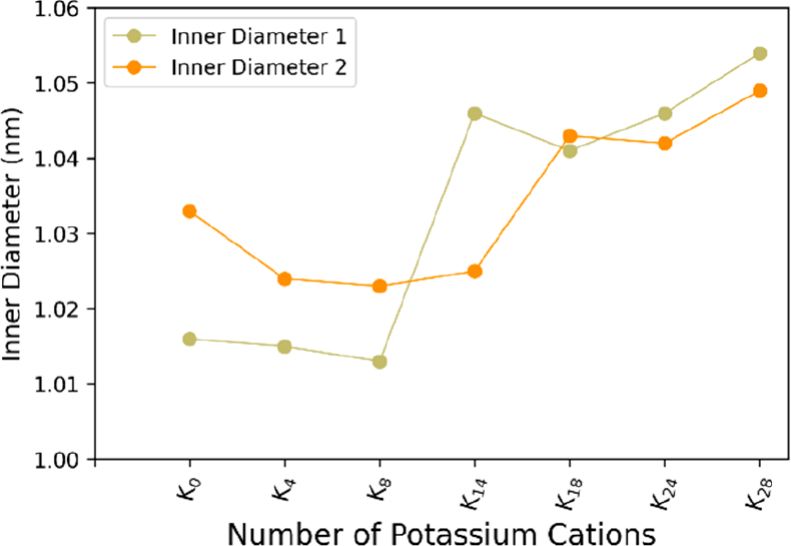
Comparison of values for the two *inner diameter* measurements in various K_*n*_[P_8_W_48_O_184_]^(40–*n*)–^ POM structures. There is a large uptick in the pore diameter between the addition of 8 and 18 Potassium cations.

This increase in the value of *inner diameter 1* actually increases symmetry throughout the POM; the “default” structure without any cations is slightly oval, with one *inner diameter* longer than the other. The addition of cations aids in decreasing the difference between these two diameter values, with a crossover point occurring between the inclusion of 8 and 14 potassium cations and the *inner diameter* values only coming within 5 pm of each other between the addition of 14 and 18 cations (Figure 7). The inclusion of cations is therefore essential for maintaining symmetry throughout the POM.

It is worth noting that cations 9–14 are added to the exterior of the POM, instead of within the pore. This arrangement of cations outside the pore as well as within is key for enforcing symmetry in a computational model, as all areas of the POM are now stabilized and prevented from being too electron dense.

This enforcement of symmetry, however, comes at the cost of increasing divergence from the empirical data. As can be seen in Figure 5, there is a difference of roughly 5 Å for the *inner diameter* and 8 Å for the outer equivalent.

While this difference is relatively small, it will likely continue to increase if one wanted to completely charge neutralize the POM or if TM oxides were bound to the framework to simulate a portion of a wider POMzite network.

Coupled with the standard increase in computational nodes and time required when one adds more atoms to a molecular system, there most likely exists a crossover point where, in most instances, the accuracy of results obtained from the model is of a suitable quality to not necessitate a more complex molecular model be utilized.

From our results, we have determined K_8_[P_8_W_48_O_184_]^32–^ to be the best all-around representation of the full K_28_H_7_Li_5_[P_8_W_48_O_184_] POM framework. It is relatively easy to converge the structure with DFT, with the computational effort rising sharply upon inclusion of additional cations, and the POM exhibits pore dimensions that are more closely in line with the empirical data than the other models we tested.

Finally, we decided to run a small experiment looking at whether countercations other than K could increase the HOMO–LUMO gap for, or otherwise via a difference in size or charge aid in stabilizing, the {P_2_W_12_} and {P_8_W_48_} POMs (see SI-13 for full results). Based on trends outlined in the literature,^1^ we expect tungsten-based POMs to be less polarized and reactive when exposed to smaller cations, thereby exhibiting a larger HOMO–LUMO gap.

Based on our models, we found that more cationic ions, such as Mg^2+^ or Ca^2+^, tended to stabilize the HOMO and LUMO orbitals relative to their monocationic counterparts, though the HOMO–LUMO gap was reduced in magnitude. We also observed that the size of the HOMO–LUMO gap decreased slightly overall as the size of the monocation increased (see SI-13, specifically Table S22 and Figure S30, for more details).

**Figure 8:**
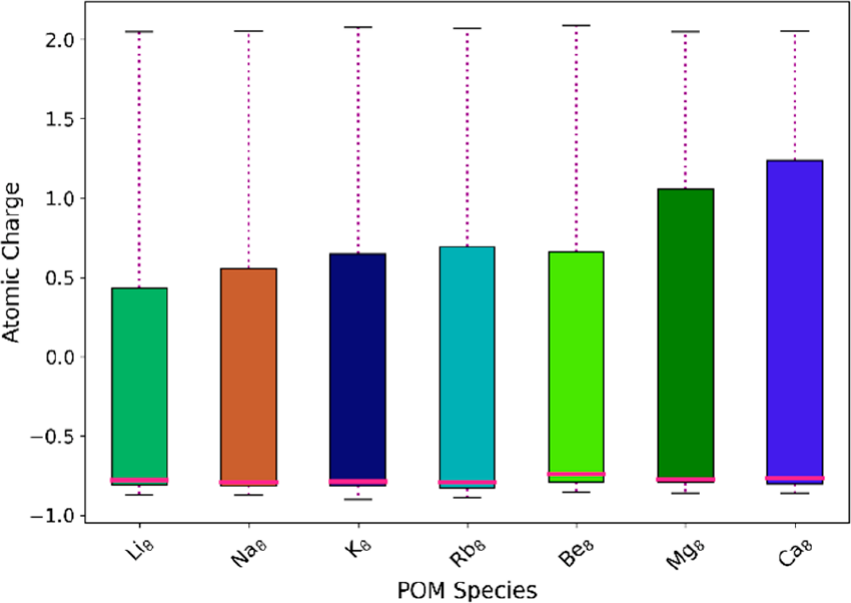
Variation in range of atomic charges within a X_8_[P_8_W_48_O_184_]^*n*−^ POM, where X = Li^+^, Na^+^, K^+^, Rb^+^, Be^2+^, Mg^2+^, Ca^2+^.

Standard deviation (STD) and mean atomic charge (MAC) calculations conducted for each POM species proved more enlightening with respect to the effect of different cation elements; the alkali metal and earth cations with the smallest ionic radius displayed the lowest SD value for the molecule as a whole and relatively low values when the constituent elements were examined individually. A small STD value in this context signifies a less polarized POM, which will, in turn, be less reactive and therefore more stable overall. MAC data similarly placed smaller cations as promoting delocalization of the framework electrons, moving the mean elemental charge toward neutral zero more than their larger counterparts. (see Figures 8 and SI-13, specifically Tables S23 and 24).

## Conclusions

In this work, we have conducted DFT-level computational research into {X_8_W_48_}-type POMs and their precursor species, WD and WD-hexalacunaries, and used the data collected to identify the structural characteristics in the molecule responsible for key chemical and electronic properties. We have also determined K_8_[P_8_W_48_O_184_]^32–^ to be the simplest representation of the full K_28_Li_5_H_7_[P_8_W_48_O_184_] molecule, while still being accurate to the empirical data present. Unless one wishes to model a POMzite subsection, for instance, or another molecular system that uses the {P_8_W_48_} POM as a building block that sees the overall anionic charge far exceed the original, already high value of −40, K_8_{P_8_W_48_} is a very suitable model. Finally, we conducted some preliminary theoretical work into the effect of using countercations other than potassium for {P_8_W_48_} POMs, with an indication that there are subtle trends down an elemental group or across a row, but overall, the presence of any countercation plays a bigger role in altering molecular properties than the species deployed. In the future, we hope to gain a better understanding of the dynamics of cation–POM interactions by studying the cation mobility on the POM surface.

## Data Availability

The data underlying this study is freely available in the ioChem-BD database at https://www.iochem-bd.org/handle/10/344938.

## Supporting Information Available

Additional experimental and computational information, including empirical data obtained from the literature and full explanations behind calculations of structural errors (PDF)

## Abbreviations

{P_2_W_18_}: the parent Wells-Dawson phosphotungstate polyoxometalate anion, with the formula [P_2_W_18_O_62_]^6–^
{P_2_W_12_}: the main [P_2_W_12_O_48_]^14–^ lacunary synthesized from the parent WD
{P_8_W_48_}: the wheel-like POM [P_8_W_48_O_184_]^40–^
K_8_{P_8_W_48_}: the full wheel with Potassium countercations present, full formula K_8_[P_8_W_48_O_184_]^32–^
{Se_8_W_48_}: [Se_8_W_48_O_176_]^32–^
{P_4_W_24_}: a half-section of {P_8_W_48_}, [P_4_W_24_O_96_]^28–^

## References

[ref1] Pope M. T. Heteropoly and Isopoly Oxometalates, 1st ed.; Springer-Verlag: Berlin, Heidelberg, 1983.

[ref2] Hill C. L. Polyoxometalates [Special Issue]. Chem. Rev. 1998, 98, 1–390. 10.1021/cr960395y. 11851497

[ref3] Pope M. T.; Muller A. Polyoxometalate Chemisty From Topology via Self-Assembly to Applications, 1st ed.; Springer Netherlands, 2001. 10.1007/0-306-47625-8

[ref4] Long D. L.; Burkholder E.; Cronin L. Polyoxometalate Clusters, Nanostructures and Materials: From Self Assembly to Designer Materials and Devices. Chem. Soc. Rev. 2007, 36 (1), 105–121. 10.1039/B502666K. 17173149

[ref5] Gumerova N. I.; Rompel A. Interweaving Disciplines to Advance Chemistry: Applying Polyoxometalates in Biology. Inorg. Chem. 2021, 60 (9), 6109–6114. 10.1021/acs.inorgchem.1c00125. 33787237 PMC8154434

[ref6] Aureliano M.; Gumerova N. I.; Sciortino G.; Garribba E.; Rompel A.; Crans D. C. Polyoxovanadates with Emerging Biomedical Activities. Coord. Chem. Rev. 2021, 447, 214143 10.1016/j.ccr.2021.214143.

[ref7] Gao Y.; Choudhari M.; Such G. K.; Ritchie C. Polyoxometalates as Chemically and Structurally Versatile Components in Self-Assembled Materials. Chem. Sci. 2022, 13 (9), 2510–2527. 10.1039/D1SC05879G. 35356680 PMC8890132

[ref8] Cameron J. M.; Guillemot G.; Galambos T.; Amin S. S.; Hampson E.; Mall Haidaraly K.; Newton G. N.; Izzet G. Supramolecular Assemblies of Organo-Functionalised Hybrid Polyoxometalates: From Functional Building Blocks to Hierarchical Nanomaterials. Chem. Soc. Rev. 2022, 51 (1), 293–328. 10.1039/D1CS00832C. 34889926

[ref9] Wang S. S.; Yang G. Y. Recent Advances in Polyoxometalate-Catalyzed Reactions. Chem. Rev. 2015, 115 (11), 4893–4962. 10.1021/cr500390v. 25965251

[ref10] Mialane P.; Mellot-Draznieks C.; Gairola P.; Duguet M.; Benseghir Y.; Oms O.; Dolbecq A. Heterogenisation of Polyoxometalates and Other Metal-Based Complexes in Metal-Organic Frameworks: From Synthesis to Characterisation and Applications in Catalysis. Chem. Soc. Rev. 2021, 50 (10), 6152–6220. 10.1039/D0CS00323A. 34027956

[ref11] Horn M. R.; Singh A.; Alomari S.; Goberna-Ferrón S.; Benages-Vilau R.; Chodankar N.; Motta N.; Ostrikov K.; Macleod J.; Sonar P.; Gomez-Romero P.; Dubal D. Polyoxometalates (POMs): From Electroactive Clusters to Energy Materials. Energy Environ. Sci. 2021, 14 (4), 1652–1700. 10.1039/D0EE03407J.

[ref12] Anjass M.; Lowe G. A.; Streb C. Molecular Vanadium Oxides for Energy Conversion and Energy Storage: Current Trends and Emerging Opportunities. Angew. Chemie - Int. Ed. 2021, 60 (14), 7522–7532. 10.1002/anie.202010577. PMC8048609 32881270

[ref13] Busche C.; Vilà-Nadal L.; Yan J.; Miras H. N.; Long D. L.; Georgiev V. P.; Asenov A.; Pedersen R. H.; Gadegaard N.; Mirza M. M.; Paul D. J.; Poblet J. M.; Cronin L. Design and Fabrication of Memory Devices Based on Nanoscale Polyoxometalate Clusters. Nature 2014, 515 (7528), 545–549. 10.1038/nature13951. 25409147

[ref14] Contant R.; Tézé A. A New Crown Heteropolyanion, K_28_Li_5_H_7_P_8_W_48_O_184_.92H_2_O: Synthesis, Structure, and Properties. Inorg. Chem. 1985, 24 (26), 4610–4614. 10.1021/ic00220a036.

[ref15] Mitchell S. G.; Gabb D.; Ritchie C.; Hazel N.; Long D. L.; Cronin L. Controlling Nucleation of the Cyclic Heteropolyanion {P_8_W_48_}: A Cobalt-Substituted Phosphotungstate Chain and Network. CrystEngComm 2009, 11 (1), 36–39. 10.1039/B813066C.

[ref16] Contant R. Inorganic Syntheses; Ginsberg A. P., Ed.; Wiley: New York, 1990.

[ref17] Boyd T.; Mitchell S. G.; Gabb D.; Long D. L.; Cronin L. Investigating Cation Binding in the Polyoxometalate-Super-Crown [P_8_W_48_O_184_]^40–^. Chem. - A Eur. J. 2011, 17 (43), 12010–12014. 10.1002/chem.201101666. 21953635

[ref18] Mal S. S.; Dickman M. H.; Kortz U.; Todea A. M.; Merca A.; Bogge H.; Glaser T.; Muller A.; Nellutla S.; Kaur N.; Van Tol J.; Dalal N. S.; Keita B.; Nadjo L. Nucleation Process in the Cavity of a 48-Tungstophosphate Wheel Resulting in a 16-Metal-Centre Iron Oxide Nanocluster. Chem. - A Eur. J. 2008, 14 (4), 1186–1195. 10.1002/chem.200701424. 18165953

[ref19] Liu G.; Liu T.; Mal S. S.; Kortz U. Wheel-Shaped Polyoxotungstate Supramolecular “Blackberry” Structure in Aqueous Solution. J. Am. Chem. Soc. 2006, 128, 10103–10110. 10.1021/ja0610840. 16881639

[ref20] Mal S. S.; Kortz U. The Wheel-Shaped Cu_20_ Tun-gstophosphate[Cu_20_Cl(OH)_24_(H_2_O)_12_(P_8_W_48_O_184_)]^25–^ Ion. Angew. Chemie - Int. Ed. 2005, 44 (24), 3777–3780. 10.1002/anie.200500682. 15887209

[ref21] Vilà-Nadal L.; Mitchell S. G.; Long D. L.; Rodríguez-Fortea A.; López X.; Poblet J. M.; Cronin L. Exploring the Rotational Isomerism in Non-Classical Wells-Dawson Anions {W_18_X}: A Combined Theoretical and Mass Spectrometry Study. Dalt. Trans. 2012, 41 (8), 2264–2271. 10.1039/C2DT11919F. 22193917

[ref22] Li S.; Zhou Y.; Ma N.; Zhang J.; Zheng Z.; Streb C.; Chen X. Organoboron-Functionalization Enables the Hierarchical Assembly of Giant Polyoxometalate Nanocapsules. Angew. Chemie - Int. Ed. 2020, 59 (22), 8537–8540. 10.1002/anie.202003550. PMC7318661 32227580

[ref23] Cameron J. M.; Gao J.; Vilà-Nadal L.; Long D. L.; Cronin L. Formation, Self-Assembly and Transformation of a Transient Selenotungstate Building Block into Clusters, Chains and Macrocycles. Chem. Commun. 2014, 50 (17), 2155–2157. 10.1039/C3CC49293A. 24424119

[ref24] Contant R.; Thouvenot R. Hétéropolyanions de Type Dawson. 2. Synthèses de Polyoxotungstoarsénates Lacunaires Dérivant de l’octadécatungstodiarsénate. Étude Structurale Par RMN Du Tungstène-183 Des Octadéca(Molybdotungstovanado)Diarsénates *Apparentés*. Can. J. Chem. 1991, 69, 1498–1506. 10.1139/v91-221.

[ref25] Lu Y.; Li Y.; Wang E.; Xu X.; Ma Y. A New Family of Polyoxometalate Compounds Built up of Preyssler Anions and Trivalent Lanthanide Cations. Inorg. Chim. Acta 2007, 360 (6), 2063–2070. 10.1016/j.ica.2006.10.045.

[ref26] Boyd T.; Mitchell S. G.; Gabb D.; Long D. L.; Song Y. F.; Cronin L. POMzites: A Family of Zeolitic Polyoxometalate Frameworks from a Minimal Building Block Library. J. Am. Chem. Soc. 2017, 139 (16), 5930–5938. 10.1021/jacs.7b01807. 28368582 PMC5423706

[ref27] Bassil B. S.; Ibrahim M.; Mal S. S.; Suchopar A.; Biboum R. N.; Keita B.; Nadjo L.; Nellutla S.; Van Tol J.; Dalal N. S.; Kortz U. Cobalt, Manganese, Nickel, and Vanadium Derivatives of the Cyclic 48-Tungsto-8-Phosphate [H_7_P_8_W_48_O_184_]^33–^. Inorg. Chem. 2010, 49 (11), 4949–4959. 10.1021/ic100050r. 20420418

[ref28] Mitchell S. G.; Boyd T.; Miras H. N.; Long D. L.; Cronin L. Extended Polyoxometalate Framework Solids: Two Mn(II)-Linked {P_8_W_48_} Network Arrays. Inorg. Chem. 2011, 50 (1), 136–143. 10.1021/ic101472s. 21117703

[ref29] Mitchell S. G.; Streb C.; Miras H. N.; Boyd T.; Long D. L.; Cronin L. Face-Directed Self-Assembly of an Electronically Active Archimedean Polyoxometalate Architecture. Nat. Chem. 2010, 2 (4), 308–312. 10.1038/nchem.581. 21124513

[ref30] Zhan C. H.; Zheng Q.; Long D. L.; Vilà-Nadal L.; Cronin L. Controlling the Reactivity of the [P_8_W_48_O_184_]^40–^ Inorganic Ring and Its Assembly into POMZite Inorganic Frameworks with Silver Ions. Angew. Chemie - Int. Ed. 2019, 58 (48), 17282–17286. 10.1002/anie.201911170. PMC6900112 31538679

[ref31] Vilà-Nadal L.; Cronin L. Design and Synthesis of Polyoxometalate-Framework Materials from Cluster Precursors. Nat. Rev. Mater. 2017, 2, 17054 10.1038/natrevmats.2017.54.

[ref32] Chen S. W.; Boubekeur K.; Gouzerh P.; Proust A. Versatile Host-Guest Chemistry and Networking Ability of the Cyclic Tungstophosphate {P_8_W_48_}: Two Further Manganese Derivatives. J. Mol. Struct. 2011, 994 (1–3), 104–108. 10.1016/j.molstruc.2011.03.003.

[ref33] Zhan C.; Cameron J. M.; Gabb D.; Boyd T.; Winter R. S.; Vilà-Nadal L.; Mitchell S. G.; Glatzel S.; Breternitz J.; Gregory D. H.; Long D. L.; MacDonell A.; Cronin L. A Metamorphic Inorganic Framework That Can Be Switched between Eight Single-Crystalline States. Nat. Commun. 2017, 8, 14185 10.1038/ncomms14185. 28194009 PMC5316803

[ref34] Bruno T. J.; Lide D. R.; Rumble J. R. CRC Handbook of Chemistry and Physics: A Ready-Reference Book of Chemical and Physical Data, 100th ed.; CRC Press, 2019.

[ref35] De La Oliva A. R.; Sans V.; Miras H. N.; Yan J.; Zang H.; Richmond C. J.; Long D. L.; Cronin L. Assembly of a Gigantic Polyoxometalate Cluster {W_200_Co_8_O_660_} in a Networked Reactor System. Angew. Chemie - Int. Ed. 2012, 51 (51), 12759–12762. 10.1002/anie.201206572. 23180577

[ref36] Zalesskiy S. S.; Kitson P. J.; Frei P.; Bubliauskas A.; Cronin L. 3D Designed and Printed Chemical Generators for on Demand Reagent Synthesis. Nat. Commun. 2019, 10 (1), 6–13. 10.1038/s41467-019-13328-6. 31792220 PMC6889270

[ref37] Kim B.; Lee S.; Kim J. Inverse Design of Porous Materials Using Artificial Neural Networks. Sci. Adv. 2020, 6 (1), eaax9324 10.1126/sciadv.aax9324. 31922005 PMC6941911

[ref38] Vilà-Nadal L. POMzites a Roadmap for Inverse Design in Metal Oxide Chemistry. Int. J. Quantum Chem. 2021, 121 (5), e26493 10.1002/qua.26493.

[ref39] Boyd T.; Mitchell S. G.; Miras H. N.; Long D. L.; Cronin L. Understanding and Mapping the Assembly of a Family of Trimeric Polyoxometalates: Transition Metal Mediated Wells-Dawson (M18)- Trimers. Dalt. Trans. 2010, 39 (28), 6460–6465. 10.1039/c002633f. 20532395

[ref40] Bergman R. G.; Danheiser R. L. Reproducibility in Chemical Research. Angew. Chemie - Int. Ed. 2016, 55 (41), 12548–12549. 10.1002/anie.201606591. 27558212

[ref41] Petrus E.; Segado M.; Bo C. Nucleation Mechanisms and Speciation of Metal Oxide Clusters. Chem. Sci. 2020, 11 (32), 8448–8456. 10.1039/D0SC03530K. 34123104 PMC8163382

[ref42] te Velde G.; Bickelhaupt F. M.; Baerends E. J.; Fonseca Guerra C.; van Gisbergen S. J. A.; Snijders J. G.; Ziegler T. J. Comput. Chem. 2001, 22, 931–967. 10.1002/jcc.1056.

[ref43] AMS 2023.1, SCM, Theoretical Chemistry, Vrije Universiteit, Amsterdam, The Netherlands. http://www.scm.com.

[ref44] Perdew J.; Burke K.; Ernzerhof M. PBE. Phys. Rev. Lett. 1996, 77, 3865–3868. 10.1103/PhysRevLett.77.3865. 10062328

[ref45] Becke A. Density-functional exchange-energy approximation with correct asymptotic behavior. Phys. Rev. A 1988, 38, 3098–3100. 10.1103/PhysRevA.38.3098. 9900728

[ref46] Perdew J. Density-functional approximation for the correlation energy of the inhomogeneous electron gas. Phys. Rev. B 1986, 33, 8822–8824. 10.1103/PhysRevB.33.8822. 9938299

[ref47] Stephens P.; Devlin F.; Chabalowski C.; Frisch M. Ab Initio Calculation of Vibrational Absorption and Circular Dichroism Spectra Using Density Functional Force Fields. J. Phys. Chem. 1994, 98, 11623–11627. 10.1021/j100096a001.

[ref48] Ernzerhof M.; Scuseria G. E. Assessment of the Perdew – Burke – Ernzerhof. J. Chem. Phys. 1999, 110, 5029–5036. 10.1063/1.478401.

[ref49] Grimme S. Accurate Description of van Der Waals Complexes by Density Functional Theory Including Empirical Corrections. J. Comput. Chem. 2004, 25 (12), 1463–1473. 10.1002/jcc.20078. 15224390

[ref50] Grimme S. Semiempirical GGA-type Density Functional Constructed with a Long-range Dispersion Correction. J. Comput. Chem. 2006, 27, 1787–1799. 10.1002/jcc.20495. 16955487

[ref51] Chai J. Da; Head-Gordon M. Systematic Optimization of Long-Range Corrected Hybrid Density Functionals. J. Chem. Phys. 2008, 128 (8), 084106–1–15. 10.1063/1.2834918. 18315032

[ref52] Zhao Y.; Truhlar D. G. A New Local Density Functional for Main-Group Thermochemistry, Transition Metal Bonding, Thermochemical Kinetics, and Noncovalent Interactions. J. Chem. Phys. 2006, 125 (19), 194101 10.1063/1.2370993. 17129083

[ref53] van Lenthe E.; Ehlers A.; Baerends E.-J. Geometry optimization in the Zero Order Regular Approximation for relativistic effects. J. Chem. Phys. 1999, 110, 8943–8953. 10.1063/1.478813.

[ref54] Sure R.; Brandenburg J. G.; Grimme S. Small Atomic Orbital Basis Set First-Principles Quantum Chemical Methods for Large Molecular and Periodic Systems: A Critical Analysis of Error Sources. ChemistryOpen 2016, 5, 94–109. 10.1002/open.201500192. 27308221 PMC4906470

[ref55] Van Lenthe E.; Baerends E. J. Optimized Slater-Type Basis Sets for the Elements 1–118. J. Comput. Chem. 2003, 24 (9), 1142–1156. 10.1002/jcc.10255. 12759913

[ref56] Klamt A. COSMO-RS From Quantum Chemistry to Fluid Phase Thermodynamics and Drug Design; Elsevier; Amsterdam, 2005.

[ref57] Pye C. C.; Ziegler T.; van Lenthe E.; Louwen J. N. An Implementation of the Conductor-like Screening Model of Solvation within the Amsterdam Density Functional Package. Part II. COSMO for Real Solvents. Can. J. Chem. 2009, 87 (7), 790–797. 10.1139/V09-008.

[ref58] Allinger N. L.; Zhou X.; Bergsma J. Molecular Mechanics Parameters. Journal of Molecular Structure THEOCHEM. 1994, 312 (1), 69–83. 10.1016/S0166-1280(09)80008-0.

[ref59] Alvarez S. A Cartography of the van Der Waals Territories. Dalt. Trans. 2013, 42 (24), 8617–8636. 10.1039/c3dt50599e. 23632803

[ref60] Miró P.; Ling J.; Qiu J.; Burns P. C.; Gagliardi L.; Cramer C. J. Experimental and Computational Study of a New Wheel-Shaped {[W_5_O_21_]_3_[(U^VI^O_2_)_2_(M-O_2_)]_3_}^30–^ Polyoxometalate. Inorg. Chem. 2012, 51, 8784–8790. 10.1021/ic3005536. 22857707

[ref61] Gulam Rabbani S. M.; Miró P. Computational Insights into Iron Heterometal Installation in Polyoxovanadate-Alkoxide Clusters. Inorg. Chem. 2023, 62, 1797 10.1021/acs.inorgchem.1c03589. 35344660

[ref62] Petrus E.; Bo C. Unlocking Phase Diagrams for Molybdenum and Tungsten Nanoclusters and Prediction of Their Formation Constants. J. Phys. Chem. A 2021, 125 (23), 5212–5219. 10.1021/acs.jpca.1c03292. 34086467

[ref63] Swart M.; Duran M.; Bickelhaupt F. M. DFT2021 Poll. Marcel Swart, 2021. https://www.marcelswart.eu/dft-poll/news2021.pdf (accessed 2 Sept 2022).

[ref64] Lapham P.; Vilà-Nadal L.; Cronin L.; Georgiev V. P. Influence of the Contact Geometry and Counterions on the Current Flow and Charge Transfer in Polyoxometalate Molecular Junctions: A Density Functional Theory Study. J. Phys. Chem. C 2021, 125 (6), 3599–3610. 10.1021/acs.jpcc.0c11038. PMC7899180 33633816

[ref65] Ly H. G. T.; Mihaylov T.; Absillis G.; Pierloot K.; Parac-Vogt T. N. Reactivity of Dimeric Tetrazirconium(IV) Wells-Dawson Polyoxometalate toward Dipeptide Hydrolysis Studied by a Combined Experimental and Density Functional Theory Approach. Inorg. Chem. 2015, 54 (23), 11477–11492. 10.1021/acs.inorgchem.5b02122. 26599585

[ref66] Ravelli D.; Dondi D.; Fagnoni M.; Albini A.; Bagno A. Predicting the UV Spectrum of Polyoxometalates by TD-DFT. J. Comput. Chem. 2011, 32 (14), 2983–2987. 10.1002/jcc.21879. 21766314

[ref67] Miró P.; Poblet J. M.; Ávalos J. B.; Bo C. Towards a Computational Treatment of Polyoxometalates in Solution Using QM Methods and Explicit Solvent Molecules. Can. J. Chem. 2009, 87 (10), 1296–1301. 10.1139/V09-059.

[ref68] Zhang F. Q.; Guan W.; Yan L. K.; Zhang Y. T.; Xu M. T.; Hayfron-Benjamin E.; Su Z. M. On the Origin of the Relative Stability of Wells-Dawson Isomers: A DFT Study of α-, β-, γ-, α*-, β*-, and γ*-[(PO_4_)_2_W_18_O_54_]^6–^ Anions. Inorg. Chem. 2011, 50 (11), 4967–4977. 10.1021/ic200203s. 21526757

[ref69] Vilà-Nadal L.; Mitchell S. G.; Markov S.; Busche C.; Georgiev V.; Asenov A.; Cronin L. Towards Polyoxometalate-Cluster-Based Nano-Electronics. Chem. - A Eur. J. 2013, 19 (49), 16502–16511. 10.1002/chem.201301631. 24281797

[ref70] Vilà-Nadal L.; Peuntinger K.; Busche C.; Yan J.; Lüders D.; Long D.; Poblet J. M.; Guldi D. M.; Cronin L. Polyoxometalate {W_18_O_56_XO_6_} Clusters with Embedded Redox-Active Main-Group Templates as Localized Inner-Cluster Radicals. Angew. Chem., Int. Ed. 2013, 52 (37), 9695–9699. 10.1002/anie.201303126. 23873517

[ref71] Cameron J. M.; Fujimoto S.; Kastner K.; Wei R. J.; Robinson D.; Sans V.; Newton G. N.; Oshio H. H. Orbital Engineering: Photoactivation of an Organofunctionalized Polyoxotungstate. Chem. - A Eur. J. 2017, 23 (1), 47–50. 10.1002/chem.201605021. 27801953

[ref72] Amin S. S.; Cameron J. M.; Winslow M.; Davies E. S.; Argent S. P.; Robinson D.; Newton G. N. A Mixed-Addenda Mo/W Organofunctionalised Hybrid Polyoxometalate. Eur. J. Inorg. Chem. 2022, 2022, e202200019 10.1002/ejic.202200019.

[ref73] López X.; Carbó J. J.; Bo C.; Poblet J. M. Structure, Properties and Reactivity of Polyoxometalates: A Theoretical Perspective. Chem. Soc. Rev. 2012, 41 (22), 7537–7571. 10.1039/c2cs35168d. 22885565

[ref74] Ammam M.; Mbomekalle I. M.; Keita B.; Nadjo L.; Fransaer J. [As_8_W_48_O_184_]^40–^, a New Crown-Shaped Heteropolyanion: Electrochemistry and Electrocatalytic Properties towards Reduction of Nitrite. Electrochim. Acta 2010, 55 (9), 3118–3122. 10.1016/j.electacta.2010.01.067.

[ref75] Mbomekallé I. M.; Bassil B. S.; Suchopar A.; Keita B.; Nadjo L.; Ammam M.; Haouas M.; Taulelle F.; Kortz U. Improved Synthesis, Structure, and Solution Characterization of the Cyclic 48-Tungsto-8-Arsenate(V), [H_4_As_8_W_48_O_184_]^36–^. J. Clust. Sci. 2014, 25 (1), 277–285. 10.1007/s10876-013-0656-2.

[ref76] Chen J. J.; Vilà-Nadal L.; Solé-Daura A.; Chisholm G.; Minato T.; Busche C.; Zhao T.; Kandasamy B.; Ganin A. Y.; Smith R. M.; Colliard I.; Carbó J. J.; Poblet J. M.; Nyman M.; Cronin L. Effective Storage of Electrons in Water by the Formation of Highly Reduced Polyoxometalate Clusters. J. Am. Chem. Soc. 2022, 144 (20), 8951–8960. 10.1021/jacs.1c10584. 35536652 PMC9171825

[ref77] Goura J.; Sundar A.; Bassil B. S.; Ćirić-Marjanović G.; Bajuk-Bogdanović D.; Kortz U. Peroxouranyl-Containing W48 Wheel: Synthesis, Structure, and Detailed Infrared and Raman Spectroscopy Study. Inorg. Chem. 2020, 59 (23), 16789–16794. 10.1021/acs.inorgchem.0c02858. 33215914

[ref78] Zhang L. C.; Xue H.; Zhu Z. M.; Zhang Z. M.; Li Y. G.; Wang E. B. Two New {P_8_W_49_} Wheel-Shaped Tungstophosphates Decorated by Co(II), Ni(II) Ions. J. Clust. Sci. 2010, 21 (4), 679–689. 10.1007/s10876-010-0286-x.

