# Supplementary material for: Computational Study into the Effects of Countercations on the [P8W48O184]40– Polyoxometalate Wheel
Source: ACS Org Inorg Au. 2023 Jul 22;3(5):274–82. doi: 10.1021/acsorginorgau.3c00014 (PMC10557121; doi:10.1021/acsorginorgau.3c00014)
Supplement: Supplementary file 1 — gg3c00014_si_001.pdf [file gg3c00014_si_001.pdf]

Supplementary Information for:

Computational Study into the Effects of Countercations on the $[P_8W_{48}O_{184}]^{40-}$ Polyoxometalate Wheel

Daniel Malcolm and Laia Vilà–Nadal*

WestCHEM, Department of Chemistry, the University of Glasgow, University Avenue, Glasgow, G12 8QQ, UK.

Table of Contents

SI−1: Benchmarking Results 3

SI−2: Alternative Se Quarters 6

SI−3: {Se_8W_{48}} and {As_8W_{48}} 7

SI−4: Hexalacunary and W_{48} Structure HOMO−LUMOs 9

SI−5: Hexalacunary Structure MEPs 10

SI−6: X_8W_{48} Wheel POM MEPs 11

SI−7: Wells−Dawson POM HOMO−LUMO 13

SI−8: Wells−Dawson POM MEPs 14

SI−9: Benchmarking {P_8W_{48}} Pore Diameter 15

SI−10: Benchmarking {As_8W_{48}} Pore Diameter 18

SI−11: Benchmarking {Se_8W_{48}} Pore Diameter 19

SI−12: K Countercations 20

SI−13: Varied Countercation Species 27

SI−1: Benchmarking Results

Table S1. List of functionals tested in this study, H-L values for the $[P_2W_{18}O_{62}]^{6-}$ Wells-Dawson. Frozen core options can be: Small (SFC), Large (LFC), or Not present (NFC)

Task	Functional	Basis Set	Frozen Core	No. of Cores (No. of Nodes)	Run Duration	Electronic Energy (Hartrees)	HOMO−LUMO (eV)
SP	PBE	TZP	SFC	20 (1)	10 minutes	−26.4997	2.32
SP	PBE	TZP	LFC	20 (1)	7 minutes	−26.0259	2.32
OPT	PBE	TZP	SFC	20 (2)	1 hour	−26.4997	2.32
OPT	PBE	TZP	LFC	20 (1)	1 hour	−26.0443	2.23
OPT	PBE	DZP	SFC	20 (1)	40 minutes	-26.4528	2.28
OPT	PBE	TZ2P	SFC	20 (1)	1 hour	−26.9075	2.43
OPT	PBE	QZ4P	SFC	20 (1)	7 hours	−26.8707	2.44
OPT	PBE−D	TZP	SFC	40 (1)	1 hour	−23.8266	0.62
OPT	PBE0	TZP	SFC	40 (1)	26 hours	−34.3282	4.34
OPT	BP86	TZP	SFC	40 (1)	35 minutes	−26.3361	2.32
OPT	BP86	TZP	LFC	20 (1)	1 hour	−25.8750	2.22
OPT	wB97x	TZP	NFC	20 (1)	72 hours	−45.8199	8.42
OPT	B3LYP	TZP	SFC	20 (2)	57 hours	−31.7635	3.82
OPT	B3LYP	TZP	LFC	20 (1)	55 hours	−31.2809	3.77
OPT	B3LYP−D	TZP	SFC	40 (1)	26 hours	−29.2849	1.13

Table S2. Comparison between HOMO and LUMO energy values reported by Vilà-Nadal *et al.*[1] and those benchmarked by ourselves.

OPT/PBE/TZP/COSMO/Small Frozen Cores

Species	Reported HOMO (eV)	Reported LUMO (eV)	Reported HOMO-LUMO (eV)	Benchmarked HOMO (eV)	Benchmarked LUMO (eV)	Benchmarked HOMO-LUMO (eV)
$[S_2Mo_{18}O_{60}]^{4-}$	-6.06	-5.18	0.88	-5.98	-5.29	0.69
$[S_2Mo_{18}O_{62}]^{4-}$	-6.77	-5.15	1.62	-6.87	-5.25	1.62
$[S_2W_{18}O_{60}]^{4-}$	-6.48	-4.61	1.87	-6.50	-4.87	1.63
$[S_2W_{18}O_{62}]^{4-}$	-6.89	-4.62	2.27	-7.12	-4.84	2.28

Table S3. Comparison between HOMO and LUMO energy values reported by Cameron *et al.*[2] (TURBOMOLE) and those benchmarked by ourselves (ADF).

SP/B3LYP/TZP/COSMO/Small Frozen Cores

Species	Reported HOMO (eV)	Reported LUMO (eV)	Reported HOMO-LUMO (eV)	Benchmarked HOMO (eV)	Benchmarked LUMO (eV)	Benchmarked HOMO-LUMO (eV)
$[P_2W_{12}O_{46}]^{14-}$	-4.41	-1.62	2.79	-3.55	-1.26	2.29
$[P_8W_{48}O_{176}]^{40-}$	-5.81	-1.97	3.83	-4.26	-1.80	2.46
$[Se_2W_{12}O_{46}]^{12-}$	-5.06	-2.16	2.90	-5.73	-1.57	4.16
$[Se_8W_{48}O_{176}]^{32-}$	-6.36	-2.53	3.82	-6.32	-2.38	3.94

Based on the results displayed in Table S2.-S4., we can conclude that ADF at the PBE level accurately describes the HOMO-LUMO energy gap and reduction energy values for POMs. Table S2. simply gave us a baseline to compare our ADF calculations with, whereas Table S3. allowed for comparison with the TURBOMOLE software; the benchmarking against TURBOMOLE is poor but this is attributed to the differences between software packages, as well as the use of B3LYP which we have found to be overestimate POM properties relative to PBE.

Table S4. Comparison between experimentally obtained HOMO and LUMO, and reduction energy values reported by Vilà-Nadal *et al.*[3] and those benchmarked by ourselves.

OPT/PBE/TZP/COSMO/Small Frozen Cores

Species	Reported HOMO-LUMO (eV)	Reported Reduction Energy (eV) (E_f^o vs NHE)	Benchmarked HOMO-LUMO (eV)	Benchmarked Reduction Energy (eV)
α-$[P_2W_{18}O_{62}]^{6-}$	2.25	-4.22	2.32	-4.08
α-$[W_{19}O_{62}]^{10-}$	1.31	-3.68	1.36	-3.15
γ*-$[W_{19}O_{62}]^{10-}$	1.60	-3.68	1.92	-3.23
γ*-$[TeW_{18}]^{10-}$	1.65	-3.36	1.95	-3.10
β*-$[IW_{18}]^{9-}$	1.04	-3.36	1.63	-4.21

For Table S4. α-isomers were in very good agreement with the experimental data but less so for the other isomers; this is attributed to the non-α-isomers being built within the ADF programme and not originating from an experimentally obtained xyz file. Manipulating structures to the extent of rotating sections tends to yield results further from the literature than those which are not.

Table S5. Comparison of bond lengths and other properties for $[P_2W_{18}O_{62}]^{6-}$ between values reported by a paper by Zhang *et al.*[4] and those benchmarked during the course of this work.

OPT/PBE/TZP/COSMO/Small Frozen Cores

Species	LUMO (eV)	P-O_i (nm)	W-O_i (nm)	W-O_t (nm)	W-O_b (nm)	P-P (nm)	W-O_e-W (°)
Reported Experimental Data	N/A	1.531-1.569	2.306-2.408	1.679-1.743	1.863-1.940	3.986	159.7-163.4
Reported Calculation Data	-4.16	1.544-1.589	2.345-2.355	1.721-1.722	1.895-1.921	3.981	161.2
Benchmarked Calculation Data	-4.38	1.556	2.400	1.733	1.934	4.000	163.2

Finally, we come to Table S5., where bond lengths and angles for the classical WD framework are benchmarked. Though our calculations are slightly out of the expected range for a couple of properties, namely P-P, and tend to be at the greater extreme of the accepted range, they are generally within the boundaries for the sake of accuracy and provide a solid end to our benchmarking.

By comparing our calculated results against reported empirical and theoretical data, we have demonstrated that PBE and TZP are the most desirable functional and basis set respectively.

SI–2: Alternative Se Quarters

Towards the beginning of this investigation we used a xyz file from a paper by Cameron J.M. *et al* for our {Se_8W_{48}} wheel.[2] It was noticed that the oxygen atom missing from the structure due to the heteroatom anion being SeO_3 (the oxygen is not missing when the anion is XO_4 or XO_6) was in a different position from that usually described for WD cages that contain a XO_3 anion (see **Fig. S1.**)[6,7,8]; unsure as to whether this was a special case or if a mistake had been made by this paper we made a geometry for the {Se_2W_{12}} where the vacant oxygen site was in the position typically assumed to be correct, and a second WD structure where the site was in the more unusual location described by the paper. The aim was to identify if one configuration of site location yielded a more stable structure and thereby elucidate which was more suitable for modelling.

Our calculations found the 'normal' configuration to be the more stable of the two, but it's worth mentioning that there is not a large difference in either the electronic energy or the size of the HOMO–LUMO gap, thus we continued to use the standard configuration for the sake of consistency.

As more hexalacunaries are synthesized and characterized, it would be prudent to determine which structure is correct when these oxo vacancies arise; it may be that the standard configuration is indeed correct and that a previously unknown rearrangement process occurs in an effort to stabilise the lacunary.

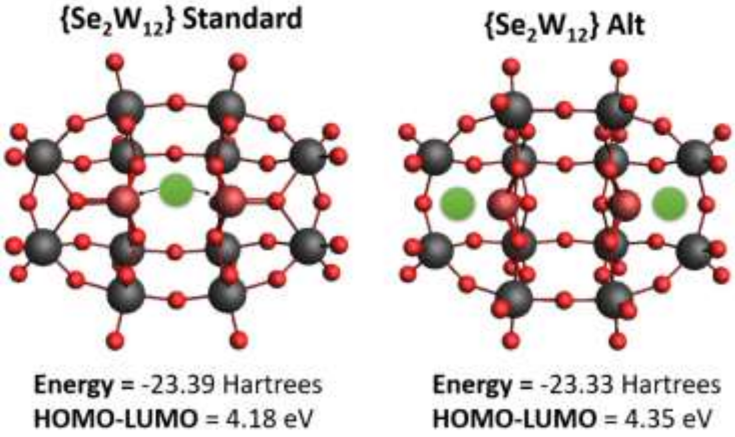

Figure S1. Comparison between 'standard' and 'alternative' {Se_2W_{12}} quarter structures. A green sphere is used to illustrate where the vacant oxygen site is in each framework. **(B3LYP/TZP/SFC/COSMO)**

SI−3: $\{Se_8W_{48}\}$ and $\{As_8W_{48}\}$

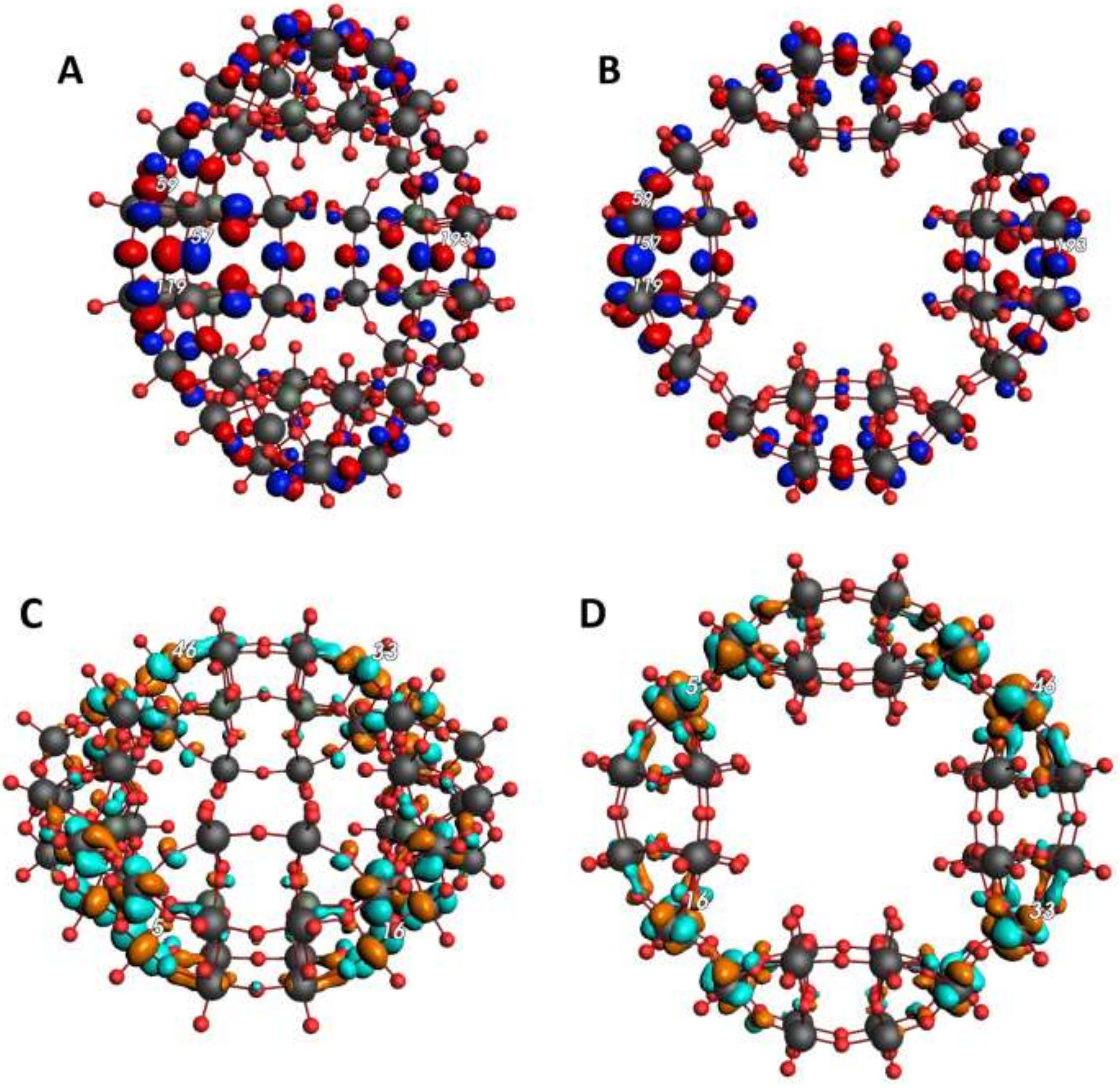

Figure S2. HOMO (**A**), (**B**) and LUMO (**C**), (**D**) visualisations for $[As_8W_{48}O_{184}]^{40-}$. The 4 atoms which contribute most to the HOMO or LUMO molecular orbitals respectively are highlighted.

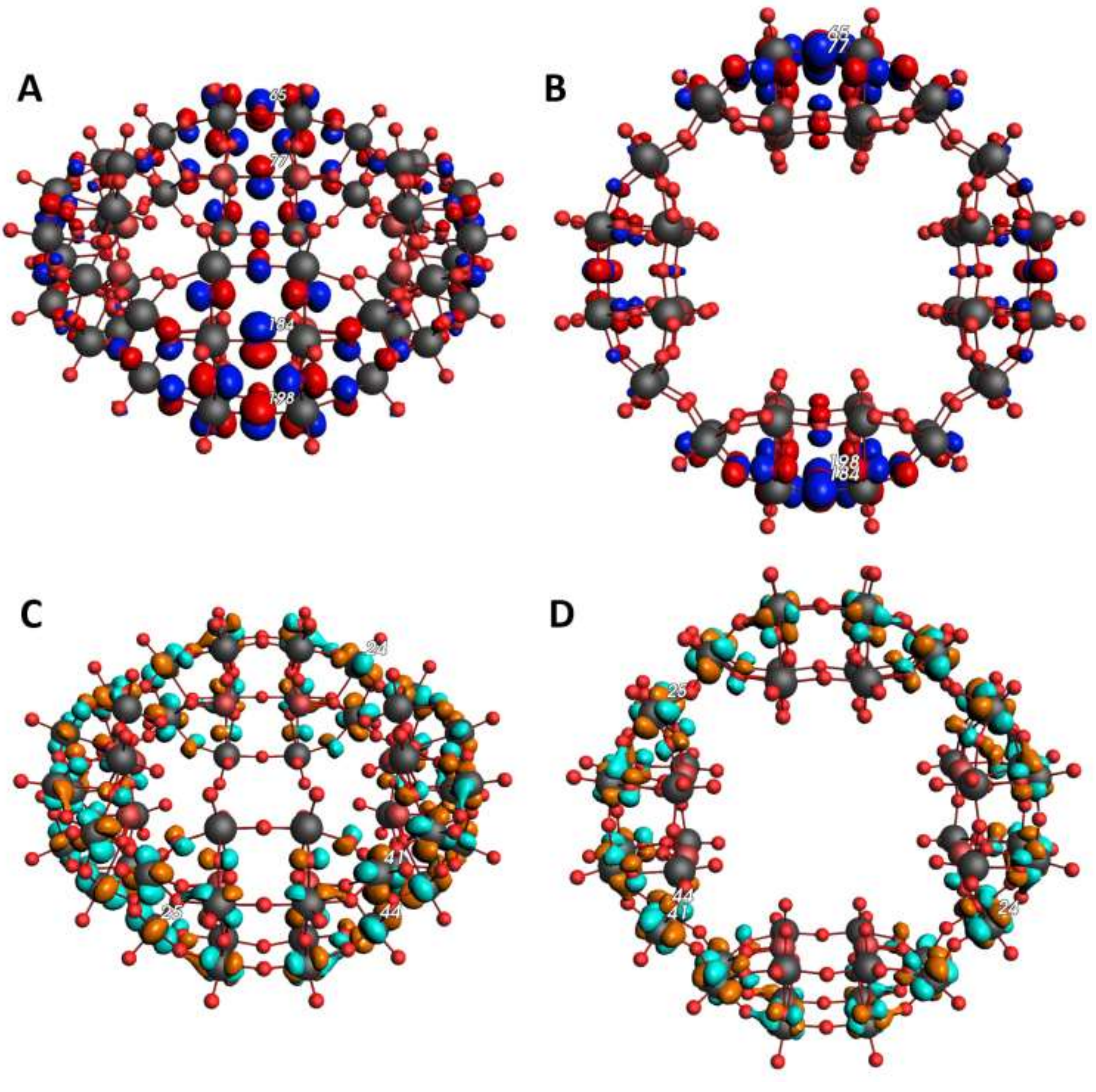

Figure S3. HOMO (**A**), (**B**) and LUMO (**C**), (**D**) visualisations for $[Se_8W_{48}O_{176}]^{32-}$. The 4 atoms which contribute most to the HOMO or LUMO molecular orbitals respectively are highlighted.

SI−4: Hexalacunary and W_{48} Structure HOMO−LUMOs

Table S6. Electronic values for different species of $[X_mW_{12}O_n]^{p-}$ lacunaries obtained with PBE functional.

PBE/TZP/COSMO/Small Frozen Cores

Species Formula	Heteroatom	E_{HOMO} (eV)	E_{LUMO} (eV)	ΔE_{H-L} (eV)
$[As_2W_{12}O_{48}]^{14-}$	AsO_4 (**V**)	−4.37	−1.57	2.80
$[P_2W_{12}O_{48}]^{14-}$	PO_4 (**V**)	−4.42	−1.60	2.82
$[Se_2W_{12}O_{46}]^{12-}$	SeO_3 (**IV**)	−4.53	−1.96	2.57
$[Se_2W_{12}O_{46}]^{12-}$ **ALT**	SeO_3 (**IV**)	−4.84	−2.05	2.79

Calculations with the **ALT** designation refer to structures described in SI−2

Table S7. Electronic values for different species of $[X_8W_{48}O_n]^{p-}$ obtained with PBE functional

PBE/TZP/COSMO/Small Frozen Cores

Species Formula	Heteroatom	E_{HOMO} (eV)	E_{LUMO} (eV)	ΔE_{H-L} (eV)
$[As_8W_{48}O_{184}]^{40-}$	AsO_4 (**V**)	-4.69	-2.06	2.62
$[P_8W_{48}O_{184}]^{40-}$	PO_4 (**V**)	−4.78	−2.17	2.61
$[Se_8W_{48}O_{176}]^{32-}$	SeO_3 (**IV**)	−5.05	−2.64	2.41

SI–5: Hexalacunary Structure MEPs

PBE/TZP/COSMO/Small Frozen Cores

$[As_2W_{12}O_{48}]^{14-}$

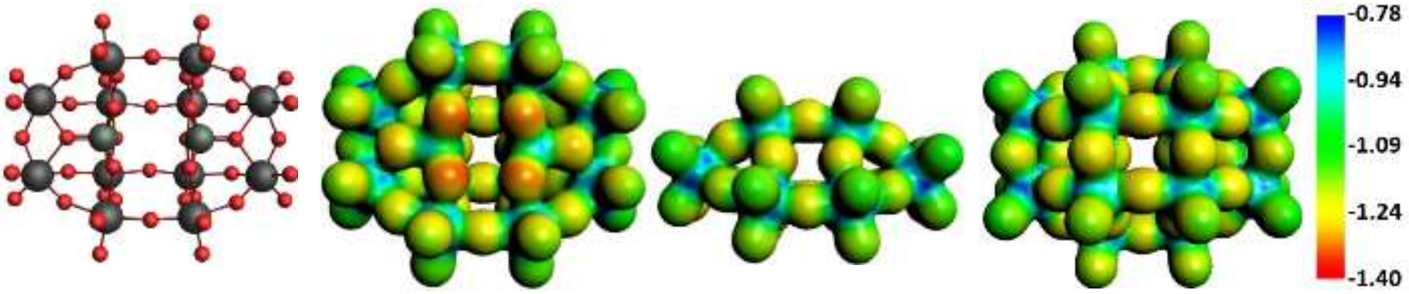

A **B** **C** **D**

Figure S4. PBE MEPs for $[As_2W_{12}O_{48}]^{14-}$ representing (**A**) Front with no MEP, (**B**) Front with MEP, (**C**) Top with MEP, and (**D**) Back with MEP. MEP sensitivity is 0.03.

$[P_2W_{12}O_{48}]^{14-}$

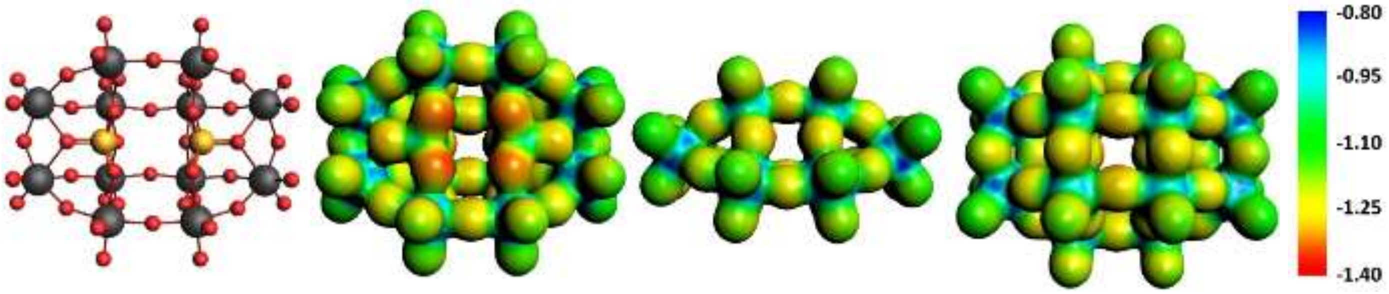

A **B** **C** **D**

Figure S5. PBE MEPs for $[P_2W_{12}O_{48}]^{14-}$ representing (**A**) Front with no MEP, (**B**) Front with MEP, (**C**) Top with MEP, and (**D**) Back with MEP. MEP sensitivity is 0.03.

$[Se_2W_{12}O_{46}]^{12-}$

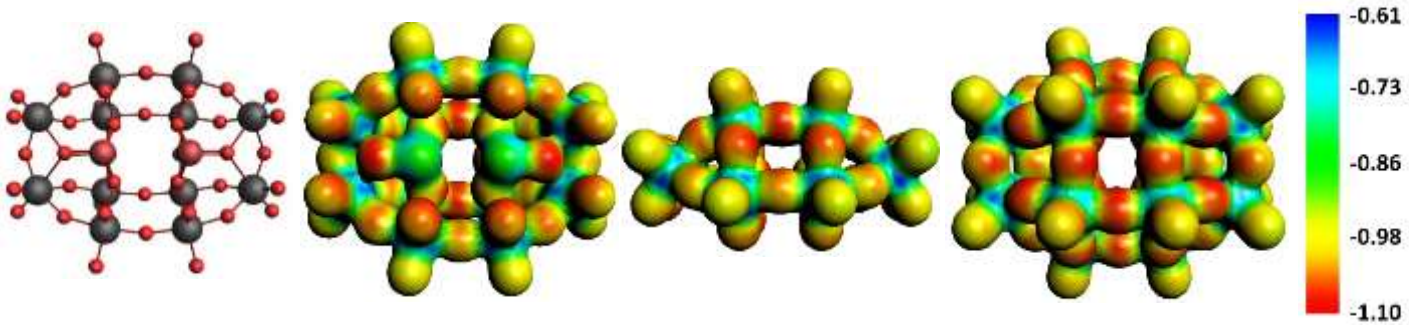

A **B** **C** **D**

Figure S6. PBE MEPs for $[Se_2W_{12}O_{46}]^{12-}$ representing (**A**) Front with no MEP, (**B**) Front with MEP, (**C**) Top with MEP, and (**D**) Back with MEP. MEP sensitivity is 0.03.

SI−6: X_8W_{48} Wheel POM MEPs

PBE/TZP/COSMO/Small Frozen Cores

$[As_8W_{48}O_{184}]^{40-}$

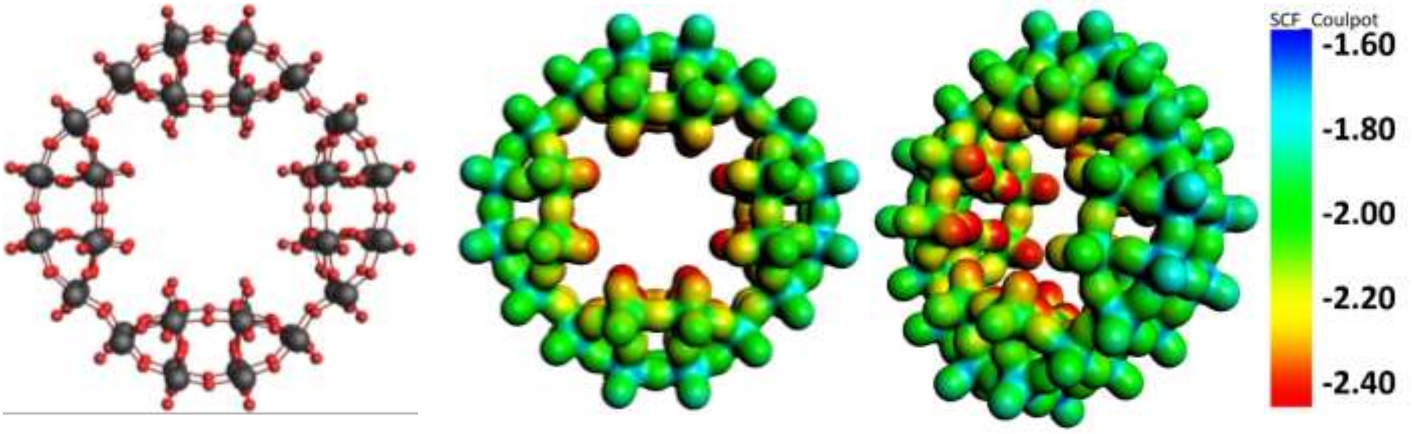

A **B** **C**

Figure S7. MEPs for $[As_8W_{48}O_{184}]^{40-}$ representing (**A**) Front with no MEP, (**B**) Front with MEP, (**C**) Angled Side view with MEP. MEP sensitivity is 0.03

$[P_8W_{48}O_{184}]^{40-}$

A **B** **C**

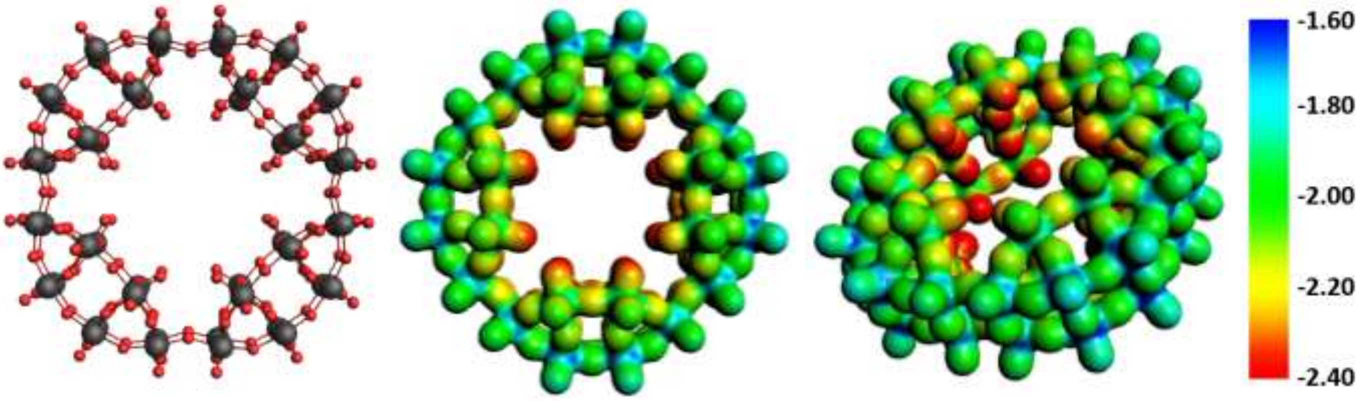

Figure S8. MEPs for $[P_8W_{48}O_{184}]^{40-}$ representing (**A**) Front with no MEP, (**B**) Front with MEP, (**C**) Angled Side view with MEP. MEP sensitivity is 0.03

$[Se_8W_{48}O_{176}]^{32-}$

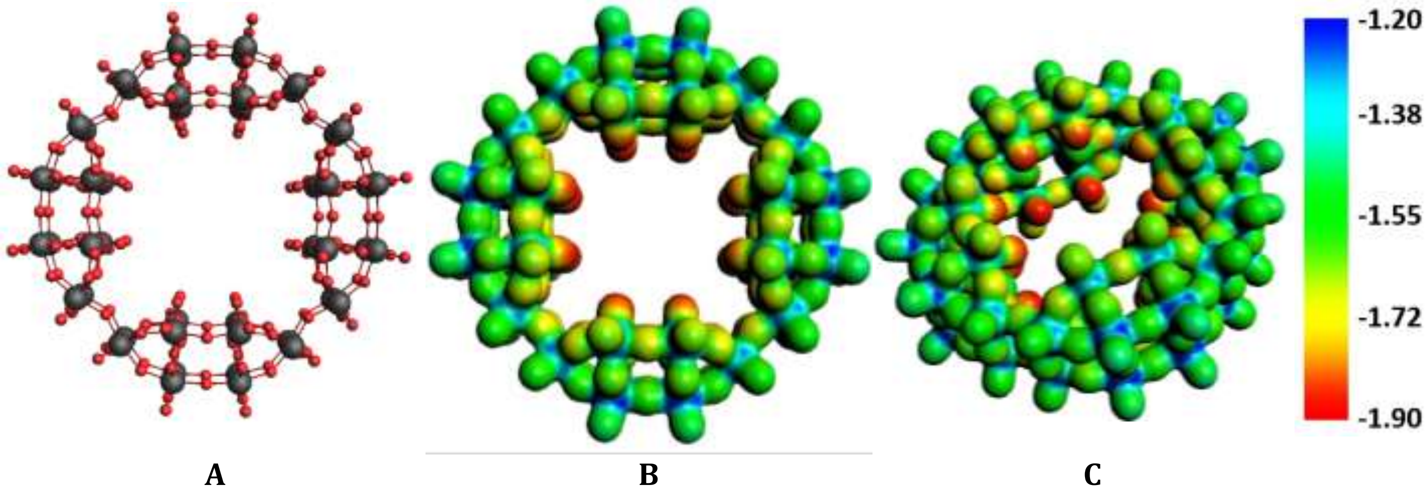

Figure S9. MEPs for $[Se_8W_{48}O_{176}]^{32-}$ representing (**A**) Front with no MEP, (**B**) Front with MEP, (**C**) Angled Side view with MEP. MEP sensitivity is 0.03.

SI–7: Wells–Dawson POM HOMO–LUMO

Table S8. Electronic values for different species of Wells–Dawsons obtained with PBE functional.

PBE/TZP/COSMO/Small Frozen Cores

Species Formula	Heteroatom	E_{HOMO} (eV)	E_{LUMO} (eV)	ΔE_{H-L} (eV)
$[As_2W_{18}O_{62}]^{6-}$	AsO_4 (**V**)	−6.72	−4.41	2.31
$[P_2W_{18}O_{62}]^{6-}$	PO_4 (**V**)	−6.70	−4.38	2.32
$[Se_2W_{18}O_{60}]^{4-}$	SeO_3 (**IV**)	−7.01	−4.91	2.10
$[Se_2W_{18}O_{60}]^{4-}$ **ALT**	SeO_3 (**IV**)	−7.12	−4.85	2.27

Calculations with the **ALT** designation refer to structures described in SI−2

SI–8: Wells–Dawson POM MEPs

PBE/TZP/COSMO/Small Frozen Cores

[$As_2W_{18}O_{62}$]$^{6-}$

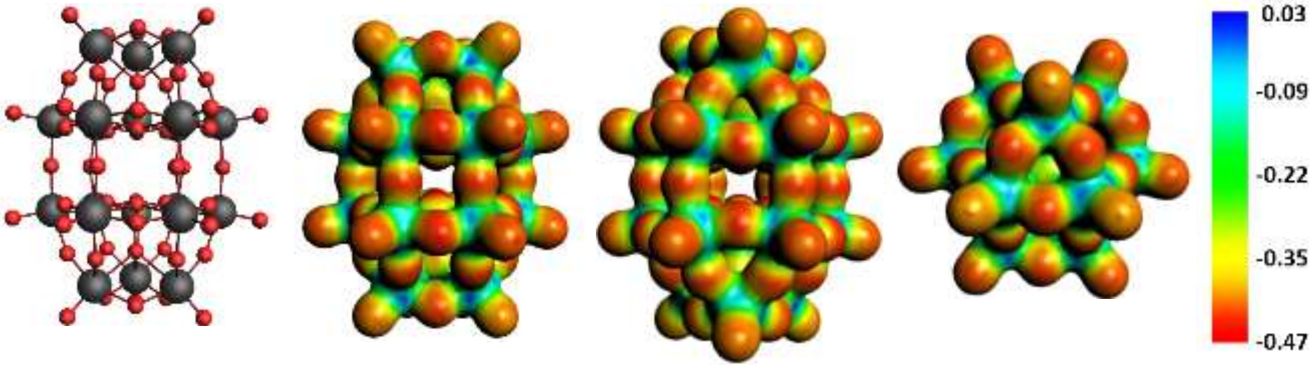

A **B** **C** **D**

Figure S10. PBE MEPs for [$As_2W_{18}O_{62}$]$^{6-}$ representing (**A**) Side 1 with no MEP, (**B**) Side 1 with MEP, (**C**) Side 2 with MEP, and (**D**) Top with MEP. MEP sensitivity is 0.03

[$P_2W_{18}O_{62}$]$^{6-}$

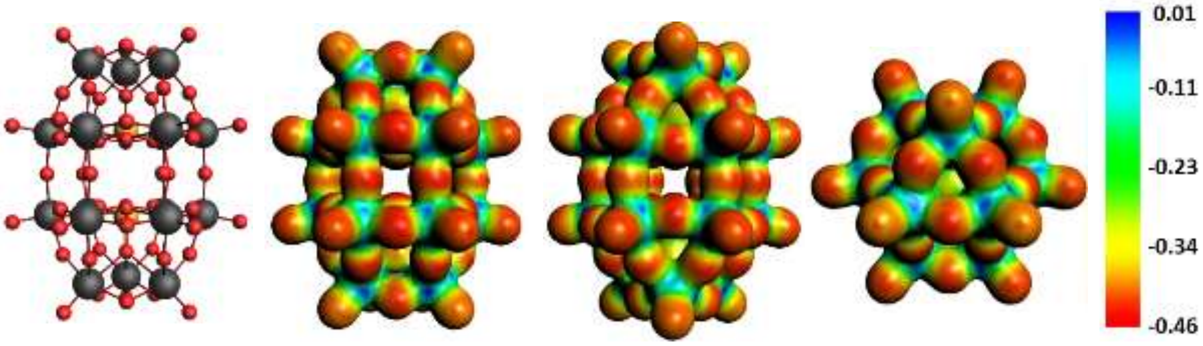

A **B** **C** **D**

Figure S11. PBE MEPs for [$P_2W_{18}O_{62}$]$^{6-}$ representing (**A**) Side 1 with no MEP, (**B**) Side 1 with MEP, (**C**) Side 2 with MEP, and (**D**) Top with MEP. MEP sensitivity is 0.037

[$Se_2W_{18}O_{60}$]$^{4-}$

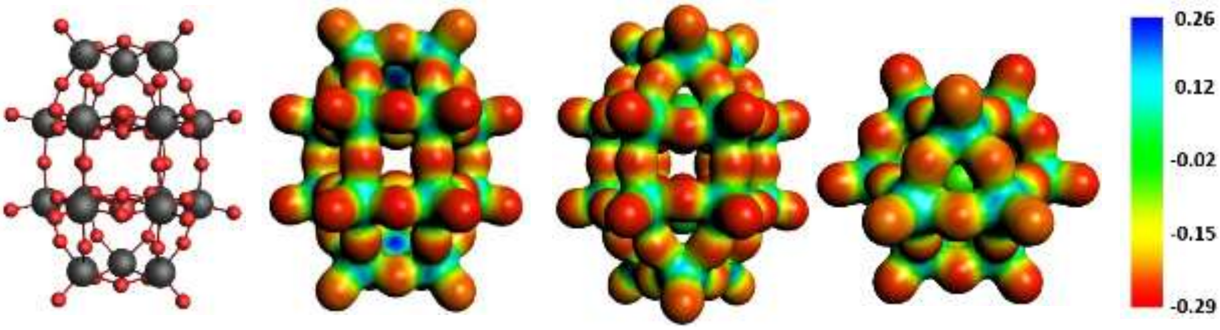

A **B** **C** **D**

Figure S12. PBE MEPs for [$Se_2W_{18}O_{60}$]$^{4-}$ representing (**A**) Side 1 with no MEP, (**B**) Side 1 with MEP, (**C**) Side 2 with MEP, and (**D**) Top with MEP. MEP sensitivity is 0.037

SI–9: Benchmarking $\{P_8W_{48}\}$ Pore Diameter

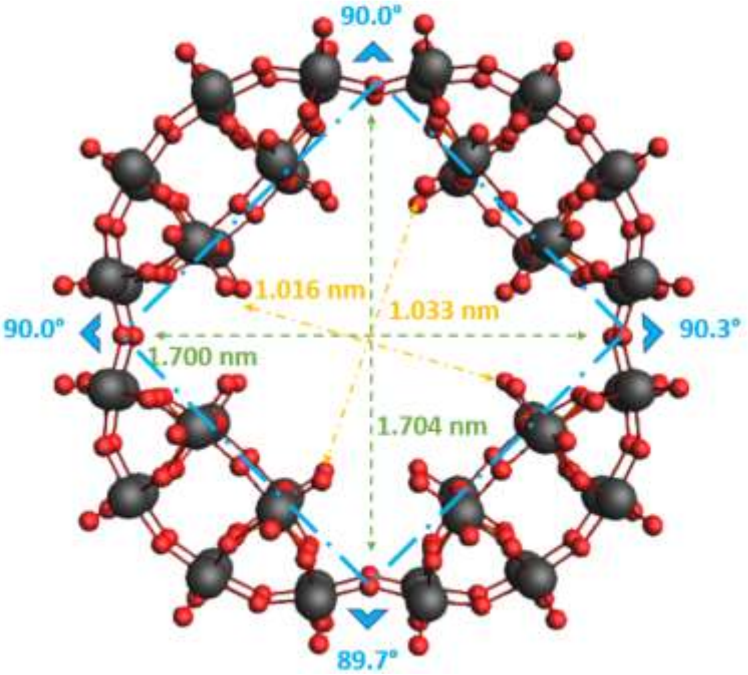

Figure S13. Structure for $[P_8W_{48}O_{184}]^{40-}$, showing measurements for angles (blue), inner diameters (yellow), and outer diameters (green).

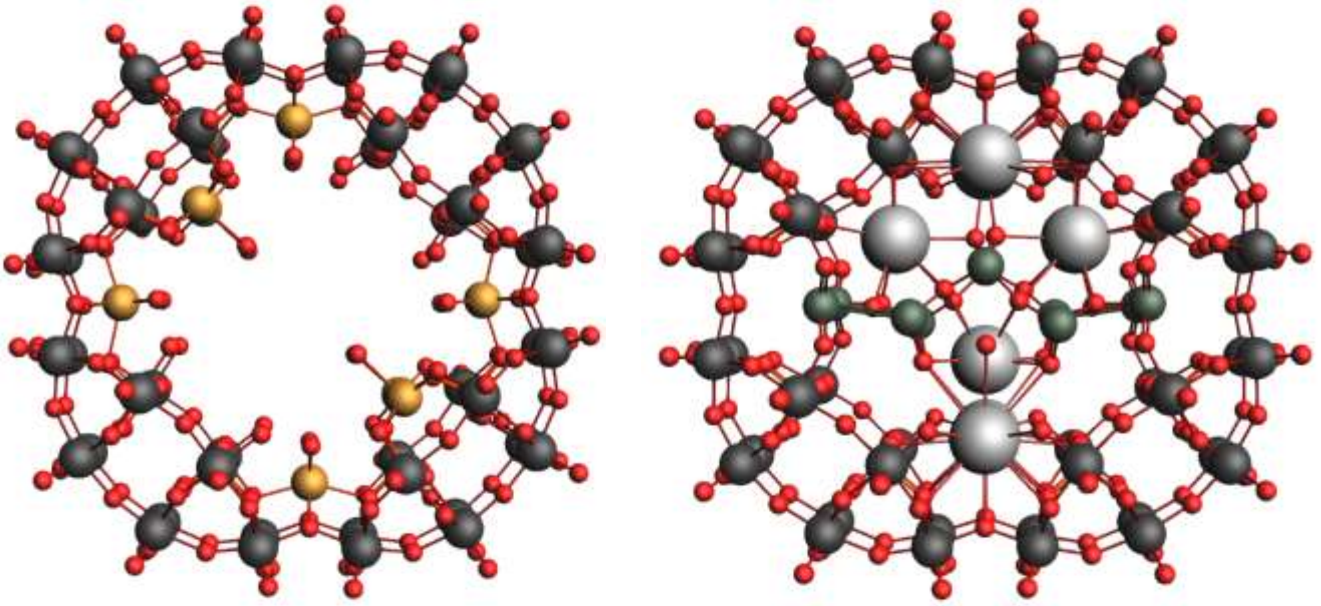

Figure S14. Geometries of $Co_8[P_8W_{48}O_{196}]^{48-}$ and $K_7As_{10}[P_8W_{48}O_{200}]^{15-}$. Cations can occupy the central pore in many different configurations, some of which, such as $K_7As_{10}[P_8W_{48}O_{200}]^{15-}$, will cause the pore to stretch to unnatural dimensions.

Table S9. Collection of empirical angle dimensions for {P_8W_{48}} structures, with a set of angles from a DFT structure for comparison. Included also is the crystal R-factor, which parameterizes the quality of the crystal.

PBE/TZP/COSMO/Small Frozen Cores

Formula	Crystal R-factor (%)	Angle 1 (°)	Angle 2 (°)	Angle 3 (°)	Angle 4 (°)	Reference
$[P_8W_{48}O_{184}]^{40-}$		90.0	90.3	89.7	90.0	Calculated
$[P_8W_{48}O_{184}]^{40-}$	4.87	91.0	88.6	91.0	88.6	*Inorg. Chem.* 2017, **56**, 22, 13822-13828
$[P_8W_{48}O_{184}]^{40-}$	6.99	90.0	90.1	89.8	90.0	*Chem. – An Asian Journal.* 2014, **9**, 2, 470-478
Mean		90.5	89.4	90.4	89.3	
$Co_8[P_8W_{48}O_{196}]^{48-}$	7.34	89.9	90.4	89.7	90.0	*Inorg. Chem.* 2019, **58**, 12, 7722-7729
$Cu_8[P_8W_{48}O_{196}]^{48-}$	7.72	89.9	90.2	89.8	90.0	*Inorg. Chem.* 2019, **58**, 12, 7722-7729
$K_7As_{10}[P_8W_{48}O_{200}]^{15-}$	7.63	81.3	98.7	81.3	98.8	*Inorg. Chem.* 2022, **61**, 51, 21024-21034
$K_8[P_8W_{48}O_{184}]^{32-}$	6.96	90.0	90.0	90.0	90.0	*Polyhedron*, 2013, **52**, 159-164
$Mn_8[P_8W_{48}O_{196}]^{48-}$	7.40	90.0	90.1	89.8	90.1	*Inorg. Chem.* 2019, **58**, 12, 7722-7729
$Ni_8[P_8W_{48}O_{196}]^{48-}$	7.33	90.0	90.4	89.5	90.1	*Inorg. Chem.* 2019, **58**, 12, 7722-7729
$Zn_8[P_8W_{48}O_{196}]^{48-}$	7.18	90.0	90.2	89.7	90.1	*Inorg. Chem.* 2019, **58**, 12, 7722-7729
Mean		88.7	91.4	88.5	91.3	

Table S10. Collection of empirical diameter dimensions for {P_8W_{48}} structures, with a set of angles from a DFT structure for comparison. Included also is the crystal R-factor, which parameterizes the quality of the crystal. **Figure S13** displays where the inner and outer ring diameters measure to and from within the pore.

PBE/TZP/COSMO/Small Frozen Cores

Formula	Inner Ring Diameter 1 (nm)	Inner Ring Diameter 2 (nm)	Outer Ring Diameter 1 (nm)	Outer Ring Diameter 2 (nm)	Reference
$[P_8W_{48}O_{184}]^{40-}$	1.016	1.033	1.700	1.704	Calculated
$[P_8W_{48}O_{184}]^{40-}$	0.966	1.035	1.632	1.667	*Inorg. Chem.* 2017, **56**, 22, 13822-13828
$[P_8W_{48}O_{184}]^{40-}$	0.977	1.017	1.644	1.646	*Chem. – An Asian Journal.* 2014, **9**, 2, 470-478
MAE	0.0445	0.0070	0.0620	0.0475	
STD	0.0055	0.0090	0.0060	0.0105	
$Co_8[P_8W_{48}O_{196}]^{48-}$	0.972	1.057	1.623	1.630	*Inorg. Chem.* 2019, **58**, 12, 7722-7729
$Cu_8[P_8W_{48}O_{196}]^{48-}$	0.978	1.069	1.617	1.620	*Inorg. Chem.* 2019, **58**, 12, 7722-7729
$K_7As_{10}[P_8W_{48}O_{200}]^{15-}$	0.932	1.094	1.790	1.536	*Inorg. Chem.* 2022, **61**, 51, 21024-21034
$K_8[P_8W_{48}O_{184}]^{32-}$	0.999	0.999	1.653	1.653	*Polyhedron*, 2013, **52**, 159-164
$Mn_8[P_8W_{48}O_{196}]^{48-}$	0.975	1.071	1.633	1.630	*Inorg. Chem.* 2019, **58**, 12, 7722-7729
$Ni_8[P_8W_{48}O_{196}]^{48-}$	0.974	1.064	1.628	1.634	*Inorg. Chem.* 2019, **58**, 12, 7722-7729
$Zn_8[P_8W_{48}O_{196}]^{48-}$	0.974	1.064	1.621	1.625	*Inorg. Chem.* 2019, **58**, 12, 7722-7729
MAE	0.1540	0.1870	0.3350	0.3000	
STD	0.2372	0.5017	0.4717	0.3735	

The TM atoms for all of the relevant structures are within the ring, facing in, leading to the increased degree of distortion in both angle and ring diameter relative to the base {P_8W_{48}} wheel (see **Fig. S14.**, where the structure of $Co_8[P_8W_{48}O_{196}]^{48-}$ is given as an example of this). $K_7As_{10}[P_8W_{48}O_{200}]^{15-}$ is a particularly strong example of this due to not only containing the greatest number of countercations out of the structures considered here, but also due to said cations forming a rigid 'core' which the POM is forced to stretch around.

SI−10: Benchmarking {As_8W_{48}} Pore Diameter

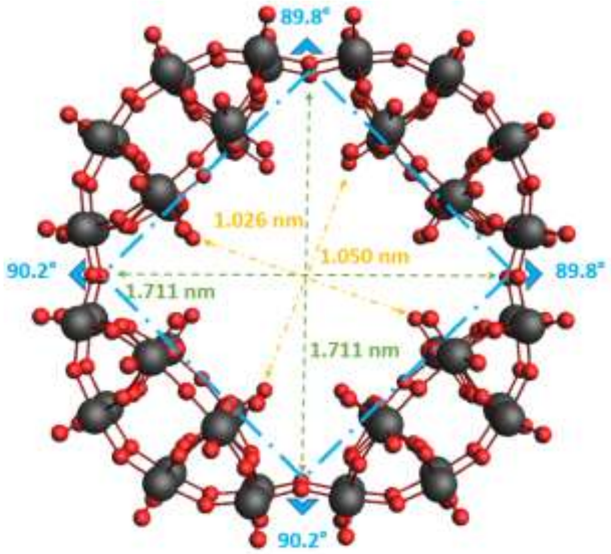

Fig S15. Structure for $[As_8W_{48}O_{184}]^{40-}$, showing measurements for angles (blue), inner diameters (yellow), and outer diameters (green).

Table S11. Collection of empirical angle dimensions for {As_8W_{48}} structures, with a set of angles from a DFT structure for comparison. Included also is the crystal R-factor, which parameterizes the quality of the crystal.

PBE/TZP/COSMO/Small Frozen Cores

Formula	Crystal R-factor (%)	Angle 1 (°)	Angle 2 (°)	Angle 3 (°)	Angle 4 (°)	Reference
$[As_8W_{48}O_{184}]^{40-}$		89.8	89.8	90.2	90.2	Calculated
$K_8[As_8W_{48}O_{184}]^{32-}$	7.74	90.0	90.0	90.0	90.0	*J. Clust. Sci.* 2014, **25**, 277-285

Table S12. Collection of empirical diameter dimensions for {As_8W_{48}} structures, with a set of angles from a DFT structure for comparison. Included also is the crystal R-factor, which parameterizes the quality of the crystal.

PBE/TZP/COSMO/Small Frozen Cores

Formula	Inner Ring Diameter 1 (nm)	Inner Ring Diameter 2 (nm)	Outer Ring Diameter 1 (nm)	Outer Ring Diameter 2 (nm)	Reference
$[As_8W_{48}O_{184}]^{40-}$	1.026	1.050	1.711	1.711	Calculated
$K_8[As_8W_{48}O_{184}]^{32-}$	1.020	1.051	1.669	1.669	*J. Clust. Sci.* 2014, **25**, 277-285

SI–11: Benchmarking {Se_8W_{48}} Pore Diameter

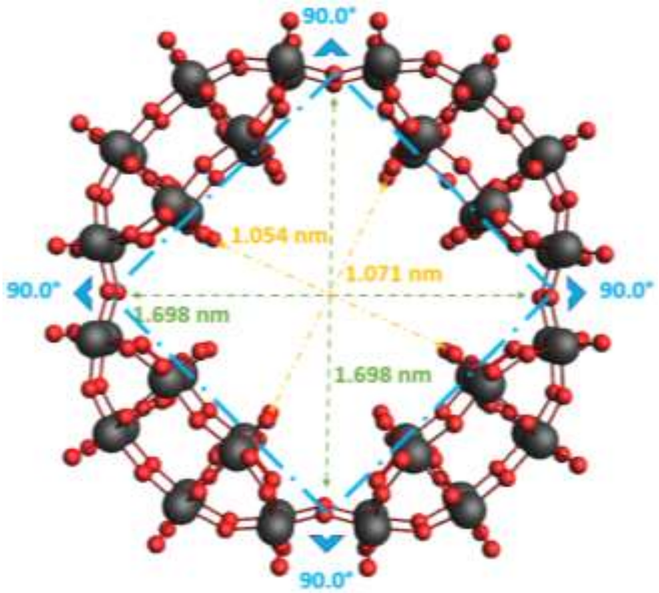

Fig S16. Structure for $[Se_8W_{48}O_{176}]^{32-}$, showing measurements for angles (blue), inner diameters (yellow), and outer diameters (green).

Table S13. Collection of empirical angle dimensions for {Se_8W_{48}} structures, with a set of angles from a DFT structure for comparison. Included also is the crystal R-factor, which parameterizes the quality of the crystal.

PBE/TZP/COSMO/Small Frozen Cores

Formula	Crystal R-factor (%)	Angle 1 (°)	Angle 2 (°)	Angle 3 (°)	Angle 4 (°)	Reference
$[Se_8W_{48}O_{176}]^{32-}$		90.0	90.0	90.0	90.0	Calculated
$[Se_8W_{48}O_{176}]^{32-}$	5.05	90.1	89.9	89.9	90.1	*Chem. Commun.* 2014, **50**, 17, 2155-2157

Table S14. Collection of empirical diameter dimensions for {Se_8W_{48}} structures, with a set of angles from a DFT structure for comparison. Included also is the crystal R-factor, which parameterizes the quality of the crystal.

PBE/TZP/COSMO/Small Frozen Cores

Formula	Inner Ring Diameter 1 (nm)	Inner Ring Diameter 2 (nm)	Outer Ring Diameter 1 (nm)	Outer Ring Diameter 2 (nm)	Reference
$[Se_8W_{48}O_{176}]^{32-}$	1.054	1.071	1.698	1.698	Calculated
$[Se_8W_{48}O_{176}]^{32-}$	1.057	1.059	1.657	1.657	*Chem. Commun.* 2014, **50**, 17, 2155-2157

SI–12: K Countercations

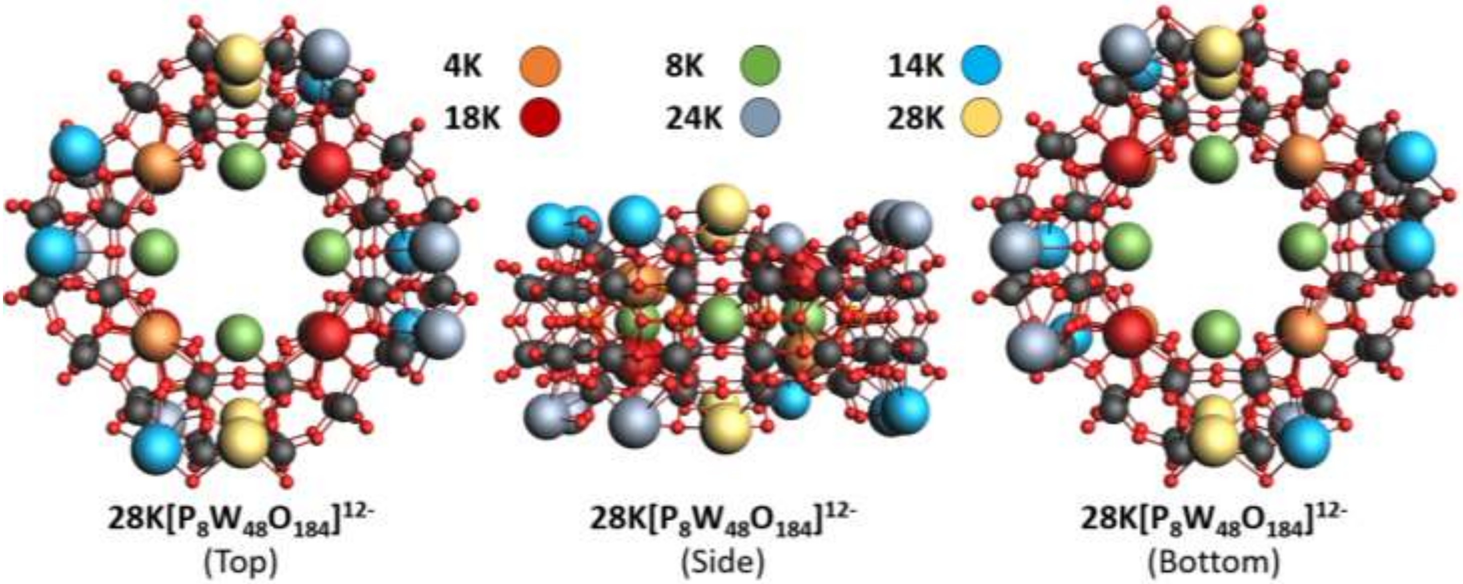

Figure S17. Geometry of $K_{28}[P_8W_{48}O_{184}]^{12-}$ with K cations coloured in order of their addition to the initial $[P_8W_{48}O_{184}]^{40-}$ structure. The orange K atoms were added first, followed by the green, and so on; in this way $K_8[P_8W_{48}O_{184}]^{32-}$ contains the atoms coloured orange and green in the above image.

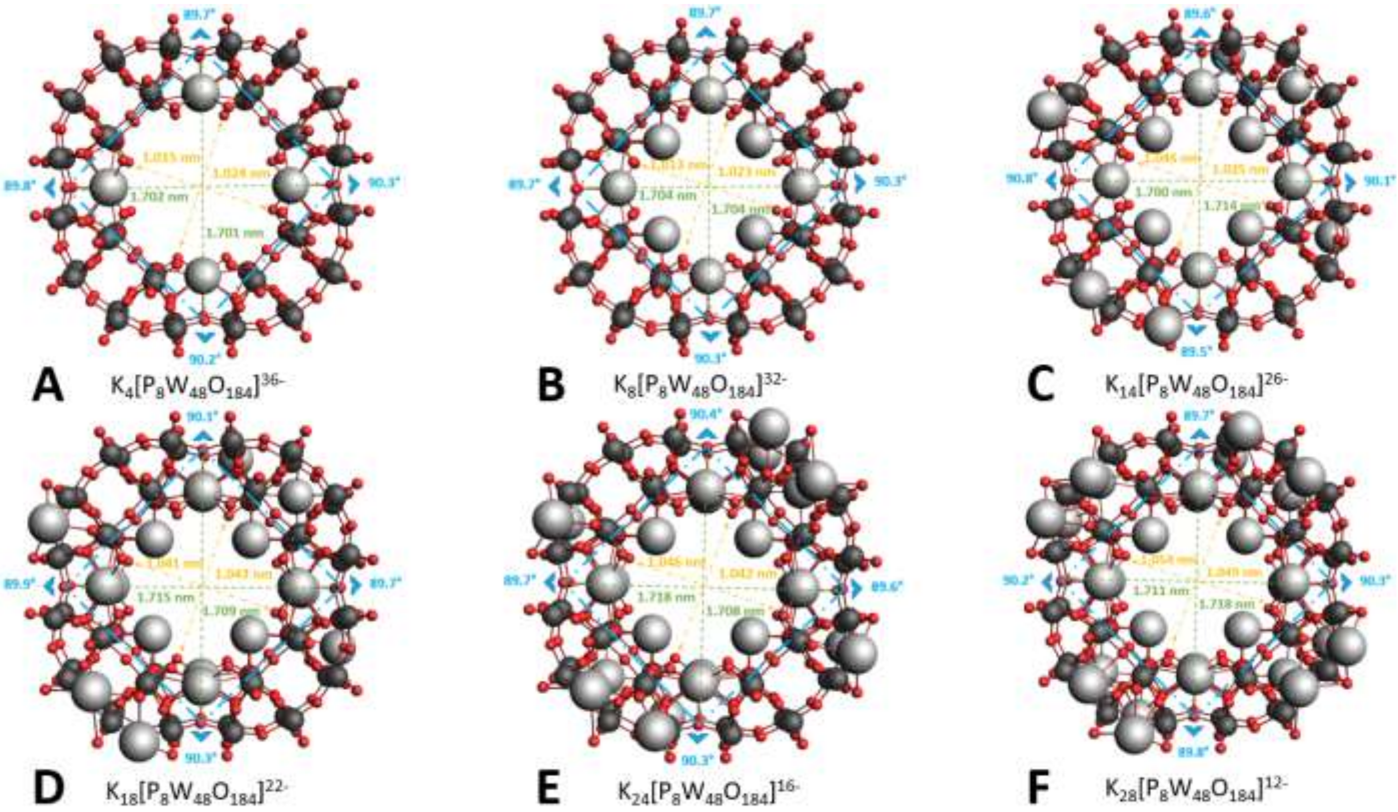

Figure S18. Stepwise addition of K cations to $\{P_8W_{48}\}$. Angles (blue), inner diameters (green), and outer diameters (yellow) are all visualized.

Table S15. Electronic values for different species of $K_n[P_8W_{48}O_{184}]^{(40-n)-}$

PBE/TZP/COSMO/Small Frozen Cores

Formula	E_{HOMO} (eV)	E_{LUMO} (eV)	ΔE_{H-L} (eV)
$[P_8W_{48}O_{184}]^{40-}$	-4.782	-2.171	2.61
$K_4[P_8W_{48}O_{184}]^{36-}$	-4.799	-2.199	2.60
$K_8[P_8W_{48}O_{184}]^{32-}$	-4.931	-2.351	2.58
$K_{14}[P_8W_{48}O_{184}]^{26-}$	-5.253	-2.567	2.69
$K_{18}[P_8W_{48}O_{184}]^{22-}$	-5.560	-2.873	2.69
$K_{24}[P_8W_{48}O_{184}]^{16-}$	-5.778	-3.119	2.66
$K_{28}[P_8W_{48}O_{184}]^{12-}$	-5.979	-3.308	2.67

Figure S19. Visualization of HOMO and LUMO stabilization as the number of K cations in the geometry

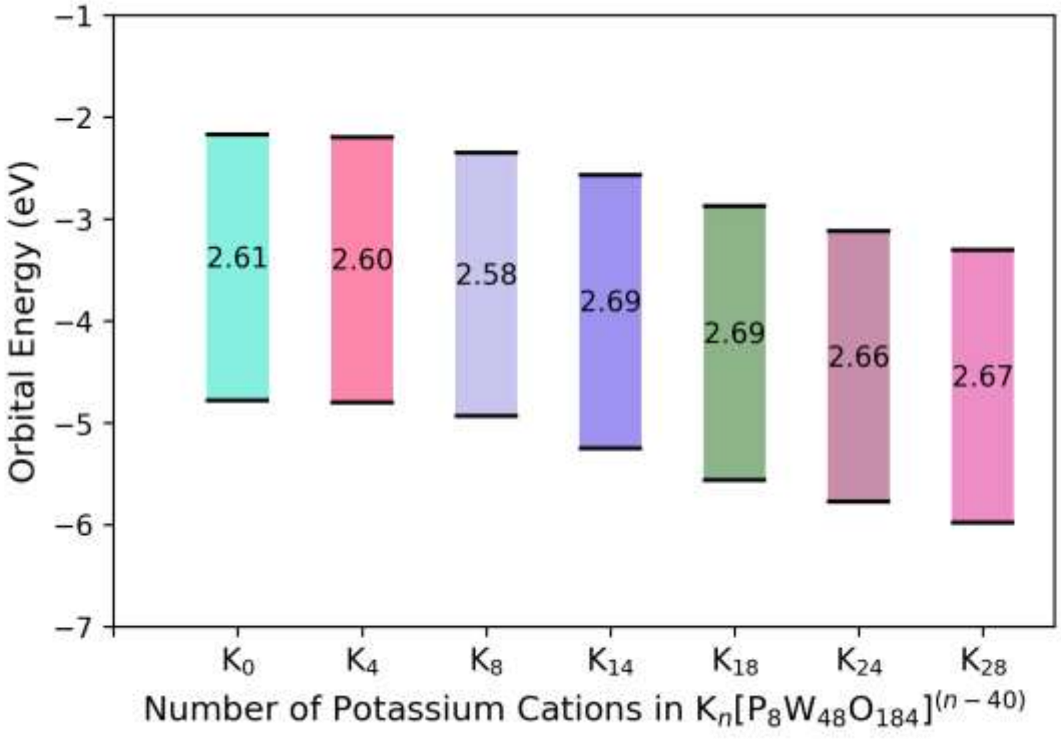

increase

Table S16. Collection of calculated angle dimensions for $K_n\{P_8W_{48}\}$ structures, with a set of empirical angles for comparison.

PBE/TZP/COSMO/Small Frozen Cores

Formula	Angle 1 (°)	Angle 2 (°)	Angle 3 (°)	Angle 4 (°)	Reference
$[P_8W_{48}O_{184}]^{40-}$	90.0	90.1	89.8	90.0	*Appl. Organomet. Chem.* 2020, **34**, 5702
$K_0[P_8W_{48}O_{184}]^{40-}$	90.0	90.3	89.7	90.0	
$K_4[P_8W_{48}O_{184}]^{36-}$	89.7	90.3	90.2	89.8	
$K_8[P_8W_{48}O_{184}]^{32-}$	89.7	90.3	90.3	89.7	
$K_{14}[P_8W_{48}O_{184}]^{26-}$	89.6	90.1	89.5	90.8	
$K_{18}[P_8W_{48}O_{184}]^{22-}$	90.1	89.7	90.3	89.9	
$K_{24}[P_8W_{48}O_{184}]^{16-}$	90.4	89.6	90.3	89.7	
$K_{28}[P_8W_{48}O_{184}]^{12-}$	89.7	90.3	89.8	90.2	

Table S17. Collection of calculated inner diameter dimensions for $K_n\{P_8W_{48}\}$ structures, with a set of empirical angles for comparison.

PBE/TZP/COSMO/Small Frozen Cores

Formula	Inner Ring Diameter 1 (nm)	Inner Ring Diameter 2 (nm)	Mean Inner Ring Diameter (nm)	Reference
$[P_8W_{48}O_{184}]^{40-}$	0.977	1.017	0.997	*Appl. Organomet. Chem.* 2020, **34**, 5702
$K_0[P_8W_{48}O_{184}]^{40-}$	1.016	1.033	1.0245	
$K_4[P_8W_{48}O_{184}]^{36-}$	1.015	1.024	1.0195	
$K_8[P_8W_{48}O_{184}]^{32-}$	1.013	1.023	1.018	
$K_{14}[P_8W_{48}O_{184}]^{26-}$	1.046	1.025	1.0355	
$K_{18}[P_8W_{48}O_{184}]^{22-}$	1.041	1.043	1.042	
$K_{24}[P_8W_{48}O_{184}]^{16-}$	1.046	1.042	1.044	
$K_{28}[P_8W_{48}O_{184}]^{12-}$	1.054	1.049	1.0515	
MAE	0.1960	0.0600	0.1280	
STD	0.6081	0.4546	0.5301	

Table S18. Collection of calculated outer diameter dimensions for $K_n\{P_8W_{48}\}$ structures, with a set of empirical angles for comparison.

PBE/TZP/COSMO/Small Frozen Cores

Formula	Outer Ring Diameter 1 (nm)	Outer Ring Diameter 2 (nm)	Mean Outer Ring Diameter (nm)	Reference
$[P_8W_{48}O_{184}]^{40-}$	1.644	1.646	1.645	*Appl. Organomet. Chem.* 2020, **34**, 5702
$K_0[P_8W_{48}O_{184}]^{40-}$	1.700	1.704	1.702	
$K_4[P_8W_{48}O_{184}]^{36-}$	1.702	1.701	1.7015	
$K_8[P_8W_{48}O_{184}]^{32-}$	1.704	1.704	1.704	
$K_{14}[P_8W_{48}O_{184}]^{26-}$	1.700	1.714	1.707	
$K_{18}[P_8W_{48}O_{184}]^{22-}$	1.715	1.709	1.712	
$K_{24}[P_8W_{48}O_{184}]^{16-}$	1.718	1.708	1.713	
$K_{28}[P_8W_{48}O_{184}]^{12-}$	1.711	1.718	1.7145	
MAE	0.2210	0.2180	0.2195	
STD	0.8862	0.8832	0.8847	

Table S19. Calculated errors between simulated and experimental data for $K_n\{P_8W_{48}\}$ structures

Formula	Mean Σ(Calc-Exp)	Mean MAE	Mean Σ(((MSE – (Calc–Exp))^2)	Mean STD
$[P_8W_{48}O_{184}]^{40-}$	0.2640	0.05107	1.2015	0.4028
$K_4[P_8W_{48}O_{184}]^{36-}$	0.2448	0.05282	1.2457	0.4101
$K_8[P_8W_{48}O_{184}]^{32-}$	0.2483	0.05382	1.2352	0.4088
$K_{14}[P_8W_{48}O_{184}]^{26-}$	0.3200	0.06307	1.1441	0.3817
$K_{18}[P_8W_{48}O_{184}]^{22-}$	0.3603	0.05982	1.0247	0.3666
$K_{24}[P_8W_{48}O_{184}]^{16-}$	0.3708	0.06182	1.0115	0.3626
$K_{28}[P_8W_{48}O_{184}]^{12-}$	0.4023	0.06282	0.9675	0.3508

As more cations are added, the model gets further away from the experimental data (larger MAE). This is associated with a decrease in the mean STD, which on paper seems to indicate better clustering around the mean; this is misleading, however. The STD trend actually relates to the calculated dimensions growing steadily away from the experimental ones, thus they become more clustered around each other relative to a further calculated value.

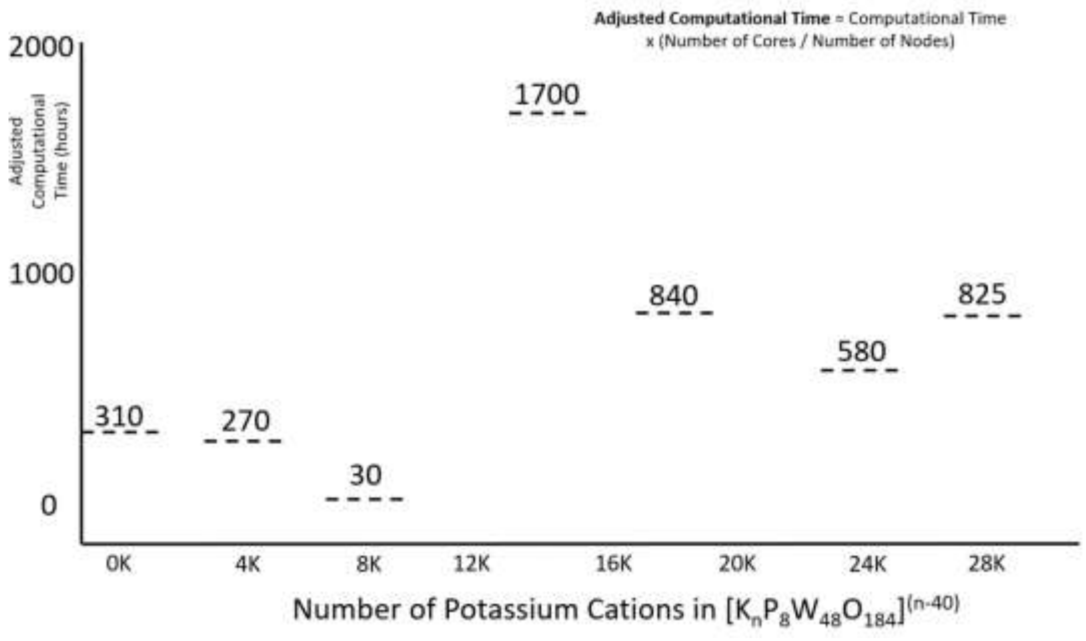

Figure S20. Trend of computational time required to converge the structure to increase as more potassium cations are included in the structure.

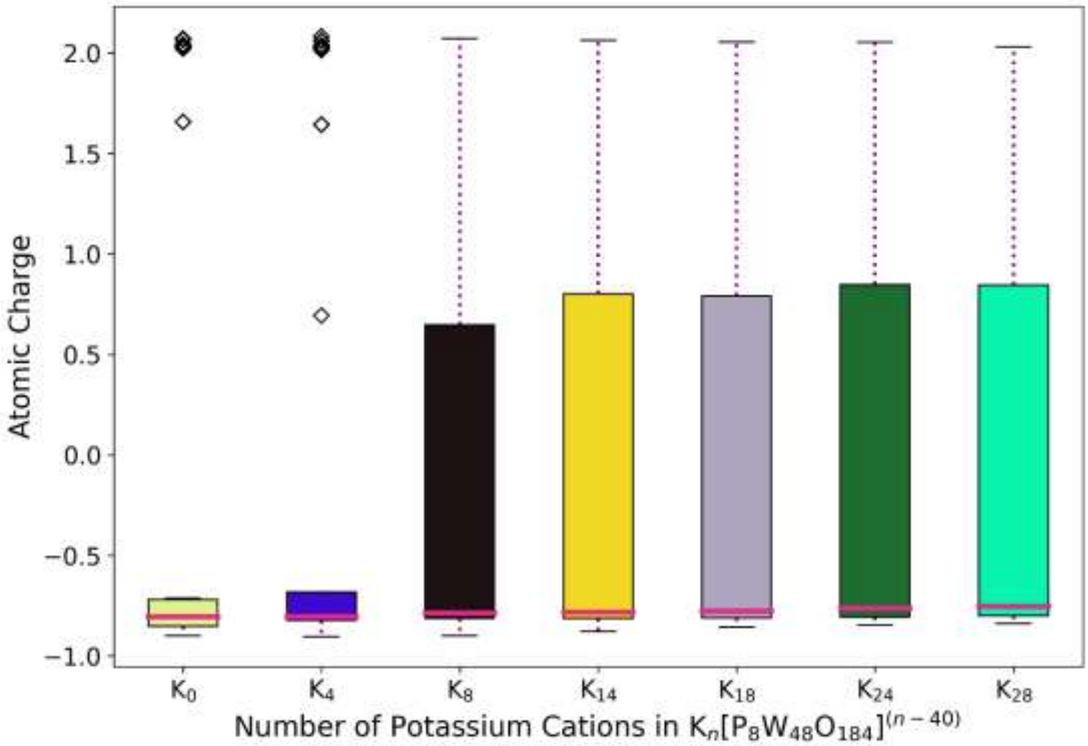

Figure S21. Difference in atomic charge throughout the whole POM species as the number of potassium cation increases.

Table S20. Standard Deviation data for $K_n[P_8W_{48}O_{184}]^{(40-n)-}$ POMs. The full formula is abbreviated to the appropriate K_n value for the structure.

Formula	K_0	K_4	K_8	K_{14}	K_{18}	K_{24}	K_{28}
Total Charge	-40.00	-36.00	-32.00	-26.00	-22.00	-16.00	-12.00
SD Whole Molecule	1.194	1.185	1.175	1.164	1.152	1.142	1.135
SD Oxygen Only	0.046	0.046	0.047	0.038	0.034	0.034	0.036
SD Tungsten Only	0.019	0.023	0.018	0.016	0.018	0.016	0.012
SD Phosphorus Only	0.000	0.001	0.001	0.002	0.003	0.003	0.003
SD Potassium Only	N/A	0.001	0.074	0.081	0.088	0.091	0.088

Standard deviation (SD) decreases throughout the entire tungsten-48 molecules as more potassium cations are added to the structure; this indicates a reduction in molecular reactivity and, therefore, an increase in stability. Examining SD by element doesn't provide much additional insight; oxygen and tungsten, the main constituent elements, become less polarised in an overall uniform manner as more cations are added. Phosphorus increases slightly, but this is due to potassium inclusion not being perfectly symmetrical with regard to these elements. It is worth noting that SD for potassium increases sharply after addition of 4 cations; this is due to a reduction in symmetry from the K_4 structure, with more potassium cations being added in increasingly individual locations in order to balance the charge.

Table S21. Mean Atomic Charge data for $K_n[P_8W_{48}O_{184}]^{(40-n)-}$ POMs. The full formula is abbreviated to the appropriate K_n value for the structure.

Formula	K_0	K_4	K_8	K_{14}	K_{18}	K_{24}	K_{28}
Total Charge	-40.00	-36.00	-32.00	-26.00	-22.00	-16.00	-12.00
Mean Atomic Charge Oxygen Only	-0.823	-0.815	-0.807	-0.800	-0.791	-0.785	-0.780
Mean Atomic Charge Tungsten Only	2.045	2.042	2.040	2.029	2.025	2.017	2.010
Mean Atomic Charge Phosphorus Only	1.658	1.644	1.609	1.612	1.598	1.597	1.598
Mean Atomic Charge Potassium Only	N/A	0.694	0.720	0.781	0.755	0.783	0.791

Compared with SD, mean atomic charge gives us a more detailed image of what occurs when the tungsten-48 POM approaches a more charge neutral state; it tells us that electron distribution becomes more evenly distributed throughout the molecule. Traditionally,

anionic oxygens become less negatively charged, whilst tungsten and phosphorus become less cationic as they accept more of the negative contribution from surrounding oxygen atoms. Potassium becomes more cationic as more countercations are added to the structure; as more cations are included, not only is the electron distribution less polarised where potassium ions are positioned throughout the structure, but each potassium also bears a smaller individual load with regards to charge balancing.

SI–13: Varied Countercation Species

PBE/TZP/COSMO/Small Frozen Cores

$Ca[P_2W_{12}O_{48}]^{12-}$

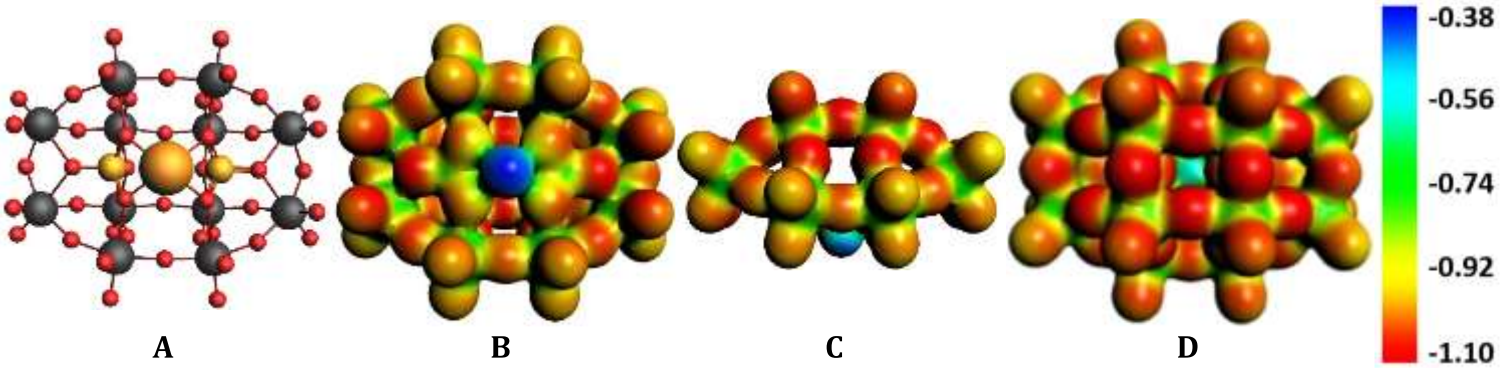

Figure S22. PBE MEPs for $Ca[P_2W_{12}O_{48}]^{12-}$ representing (**A**) Front with no MEP, (**B**) Front with MEP, (**C**) Top with MEP, and (**D**) Back with MEP. MEP sensitivity is 0.03.

$Cs[P_2W_{12}O_{48}]^{13-}$

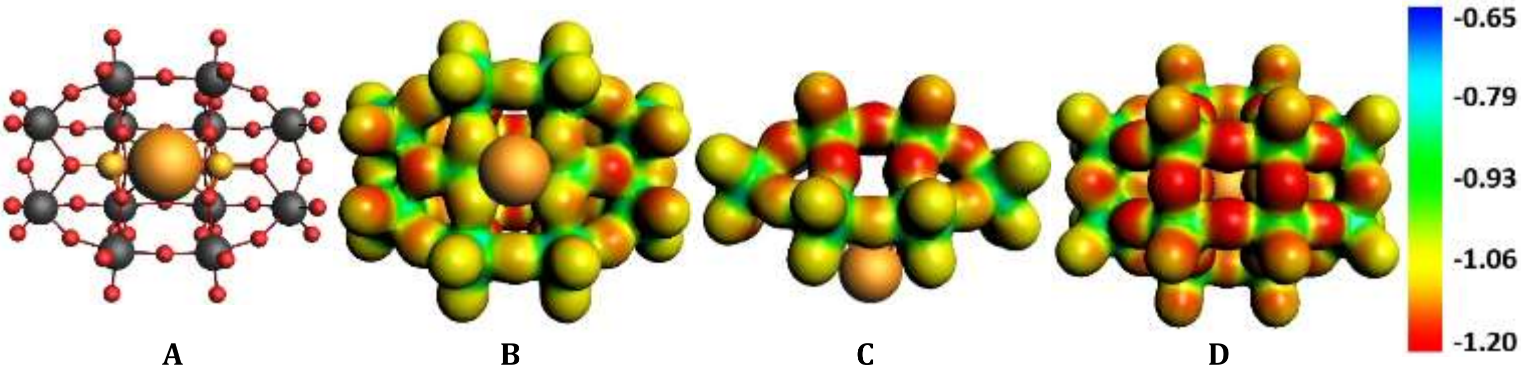

Figure S23. PBE MEPs for $Cs[P_2W_{12}O_{48}]^{13-}$ representing (**A**) Front with no MEP, (**B**) Front with MEP, (**C**) Top with MEP, and (**D**) Back with MEP. MEP sensitivity is 0.03.

$Fe[P_2W_{12}O_{48}]^{11-}$

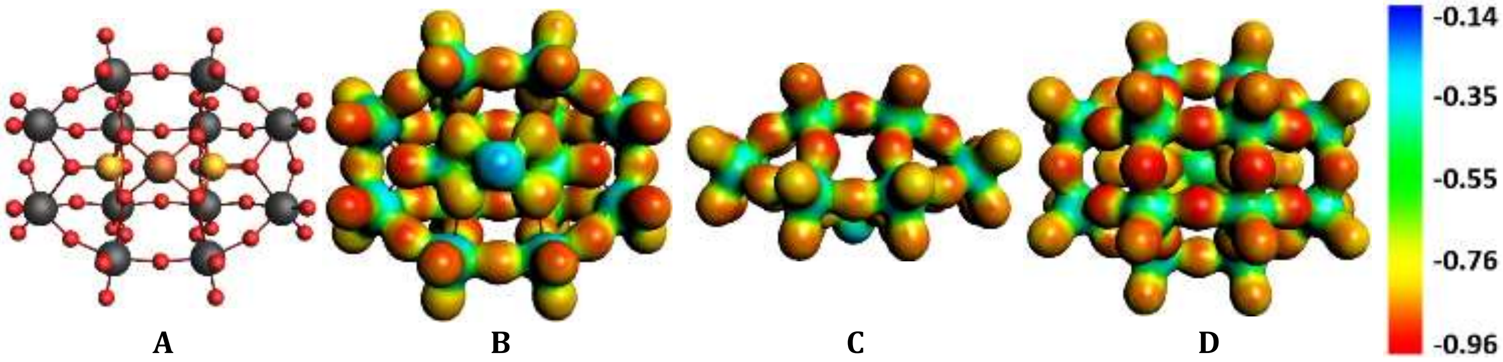

Figure S24. PBE MEPs for $Cs[P_2W_{12}O_{48}]^{13-}$ representing (**A**) Front with no MEP, (**B**) Front with MEP, (**C**) Top with MEP, and (**D**) Back with MEP. MEP sensitivity is 0.03.

$K[P_2W_{12}O_{48}]^{13-}$

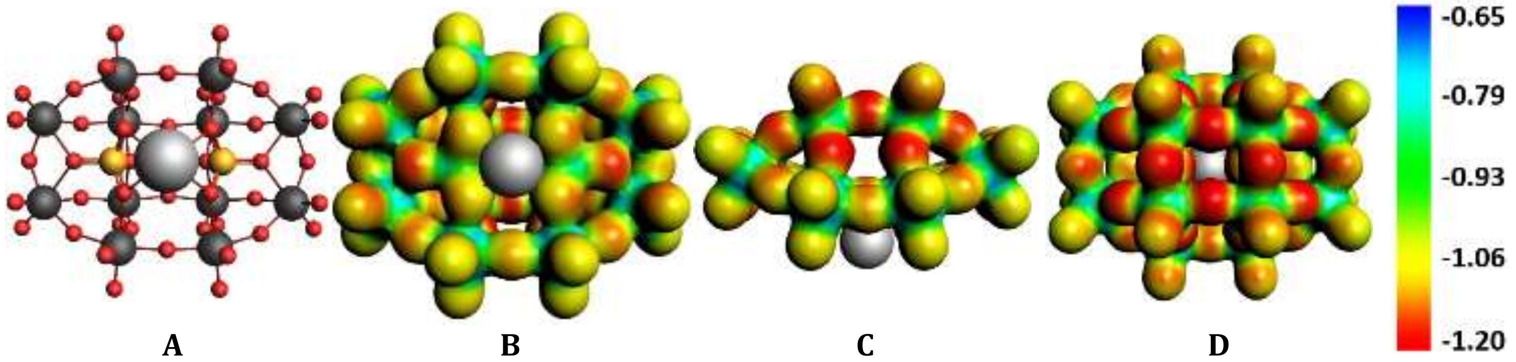

Figure S25. PBE MEPs for $K[P_2W_{12}O_{48}]^{13-}$ representing (**A**) Front with no MEP, (**B**) Front with MEP, (**C**) Top with MEP, and (**D**) Back with MEP. MEP sensitivity is 0.037.

$Li[P_2W_{12}O_{48}]^{13-}$

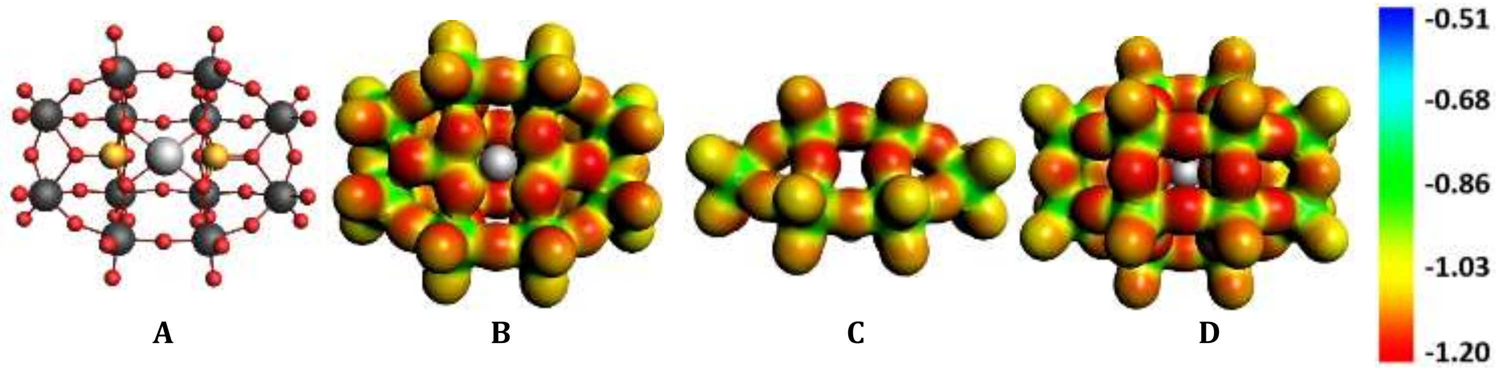

Figure S26. PBE MEPs for $Li[P_2W_{12}O_{48}]^{13-}$ representing (**A**) Front with no MEP, (**B**) Front with MEP, (**C**) Top with MEP, and (**D**) Back with MEP. MEP sensitivity is 0.037.

$Mg[P_2W_{12}O_{48}]^{12-}$

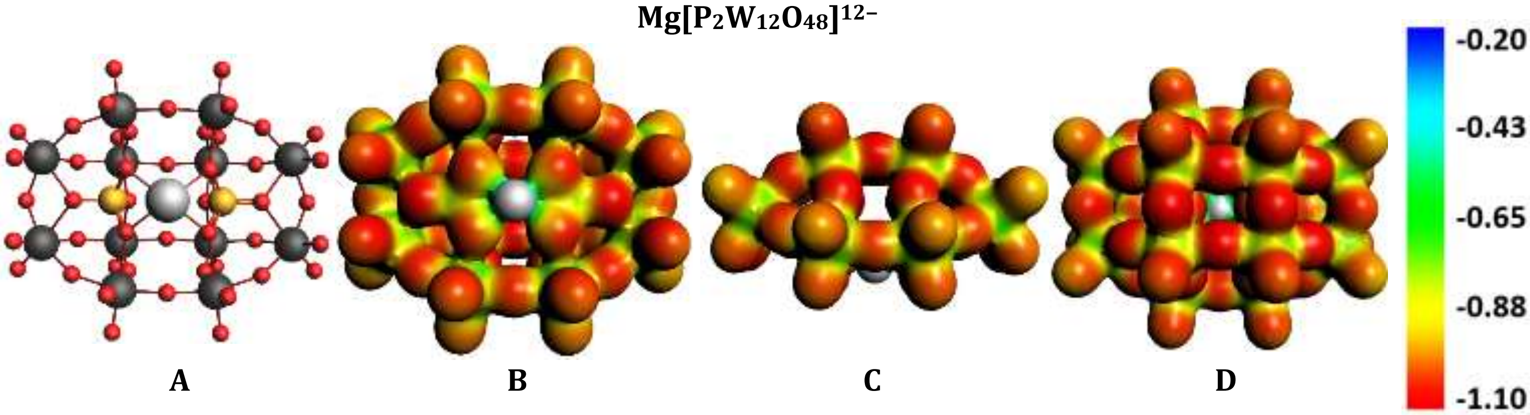

Figure S27. PBE MEPs for $Mg[P_2W_{12}O_{48}]^{12-}$ representing (**A**) Front with no MEP, (**B**) Front with MEP, (**C**) Top with MEP, and (**D**) Back with MEP. MEP sensitivity is 0.03.

$Na[P_2W_{12}O_{48}]^{13-}$

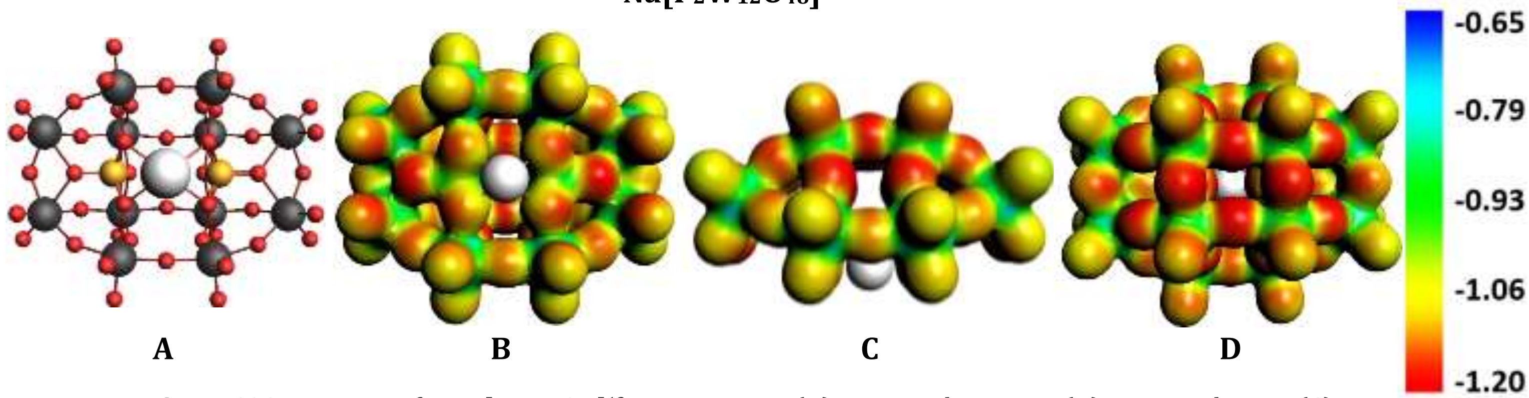

Figure S28. PBE MEPs for $Na[P_2W_{12}O_{48}]^{13-}$ representing (**A**) Front with no MEP, (**B**) Front with MEP, (**C**) Top with MEP, and (**D**) Back with MEP. MEP sensitivity is 0.03.

$Rb[P_2W_{12}O_{48}]^{13-}$

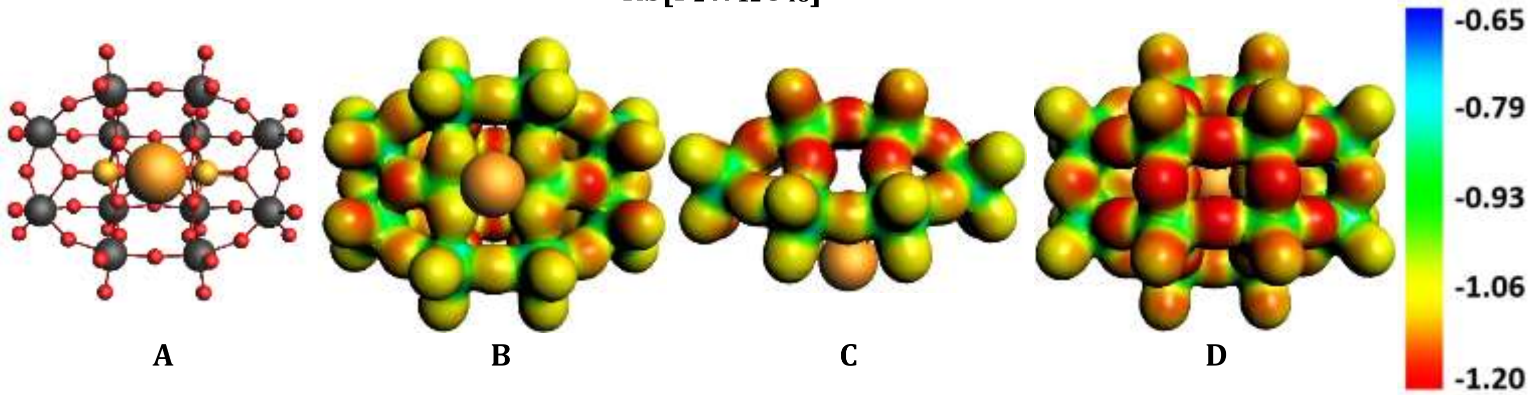

Figure S29. PBE MEPs for $Rb[P_2W_{12}O_{48}]^{13-}$ representing (**A**) Front with no MEP, (**B**) Front with MEP, (**C**) Top with MEP, and (**D**) Back with MEP. MEP sensitivity is 0.03.

Table S22. Electronic values for different species of $X_8[P_8W_{48}O_{184}]^{n-}$

PBE/TZP/COSMO/Small Frozen Cores

Formula	E_{HOMO} (eV)	E_{LUMO} (eV)	ΔE_{H-L} (eV)
$Be_8[P_8W_{48}O_{184}]^{24-}$	-5.823	-3.404	2.42
$Ca_8[P_8W_{48}O_{184}]^{24-}$	-5.722	-3.159	2.56
$K_8[P_8W_{48}O_{184}]^{32-}$	-4.931	-2.351	2.58
$Li_8[P_8W_{48}O_{184}]^{32-}$	-5.212	-2.528	2.68
$Mg_8[P_8W_{48}O_{184}]^{24-}$	-5.806	-3.364	2.44
$Na_8[P_8W_{48}O_{184}]^{32-}$	-5.163	-2.437	2.73
$Rb_8[P_8W_{48}O_{184}]^{32-}$	-5.041	-2.461	2.58

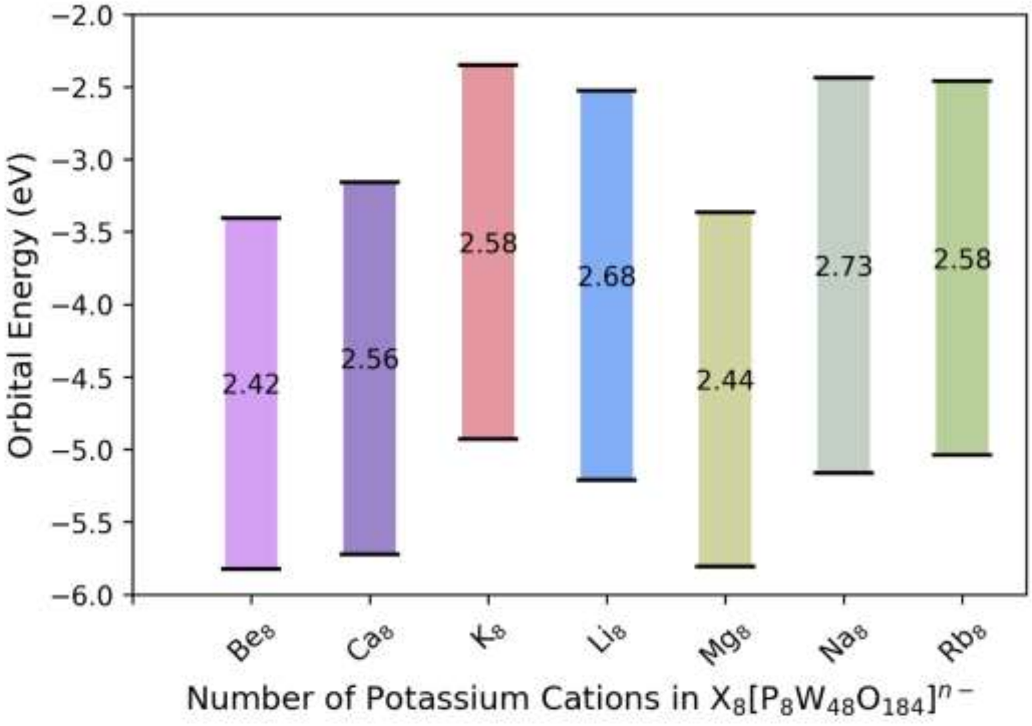

Figure S30. Visualization of variations in HOMO and LUMO stabilization as the identity of countercation in a $\{P_8W_{48}\}$-type POM changes.

Table S23. Standard Deviation (STD) values for $X_8[P_8W_{48}O_{184}]^{n-}$-type POMs. Each POM is abbreviated to only show the X_n countercation for that specific framework.

**Formula**	**No Cation**	**Li_8**	**Na_8**	**K_8**	**Rb_8**	**Be_8**	**Mg_8**	**Ca_8**
Total Charge	-40.00	-32.00	-32.00	-32.00	-32.00	-24.00	-24.00	-24.00
STD Whole Molecule	1.194	1.161	1.167	1.175	1.177	1.152	1.173	1.185
STD Oxygen Only	0.046	0.044	0.040	0.047	0.042	0.051	0.038	0.041
STD Tungsten Only	0.019	0.013	0.013	0.018	0.017	0.018	0.017	0.015
STD Phosphorus Only	0.000	0.002	0.001	0.001	0.001	0.473	0.003	0.000
STD 'X' Cation Only	N/A	0.032	0.085	0.074	0.061	0.025	0.120	0.124

The STD values for tungsten-48 POMs containing a range of different elemental countercations correlate well with the literature in 2 key points; cations are required to stabilise the highly anionic POM wheel, see that the greatest STD value for the whole framework is for the POM with no cations present, and smaller cations are the most effective at stabilizing the structure as they exhibit the largest charge density. This last point is represented by the tendency of smaller cations to trigger precipitation of the POM out of solution.

A lot of the difference in STD within the framework is tied to variation in the element used as cation. The dicationic species in particular have relatively high variance between individual atoms, which may explain why the HOMO-LUMO gaps for these POMs are smaller than their monocationic counterparts.

It is worth mentioning that $Be_8[P_8W_{48}O_{184}]^{24-}$ has a strained structure, lacking the ordered symmetry of the other POM frameworks. This may be due to Be being the smallest cation experimented with but regardless, it displays relatively anomalous results, such as a STD value for the phosphorus heteroatom that is two orders of magnitude bigger than the same property from the other POMs.

Table S24. Mean atomic charge values for $X_8[P_8W_{48}O_{184}]^{n-}$-type POMs. Each POM is abbreviated to only show the X countercation, which varies between POMs, for that specific framework.

Formula	No Cation	Li_8	Na_8	K_8	Rb_8	Be_8	Mg_8	Ca_8
Total Charge	-40.00	-32.00	-32.00	-32.00	-32.00	-24.00	-24.00	-24.00
Mean Atomic Charge Oxygen Only	-0.823	-0.794	-0.802	-0.807	-0.809	-0.761	-0.781	-0.790
Mean Atomic Charge Tungsten Only	2.045	2.033	2.033	2.040	2.040	2.037	2.035	2.036
Mean Atomic Charge Phosphorus Only	1.658	1.597	1.602	1.609	1.623	1.135	1.575	1.586
Mean Atomic Charge 'X' Cation Only	N/A	0.466	0.638	0.720	0.754	0.677	1.173	1.360

The data for mean atomic charge highlights how the smaller cation has a more stabilizing effect on the POM; $Li_8[P_8W_{48}O_{184}]^{32-}$ is the best example of this, with all the mean atomic charge values for the various elements being collectively closer to zero than the other examples.

Table S25. Collection of calculated angle dimensions for $K_n\{P_8W_{48}\}$ structures, with a set of empirical angles for comparison.

Formula	Angle 1 (°)	Angle 2 (°)	Angle 3 (°)	Angle 4 (°)	Reference
$[P_8W_{48}O_{184}]^{40-}$	90.0	90.1	89.8	90.0	*Appl. Organomet. Chem.* 2020, **34**, 5702
$Li_8[P_8W_{48}O_{184}]^{32-}$	89.5	90.0	90.1	90.5	
$Na_8[P_8W_{48}O_{184}]^{32-}$	90.3	89.9	90.0	89.8	
$K_8[P_8W_{48}O_{184}]^{32-}$	89.7	90.3	90.3	89.7	
$Rb_8[P_8W_{48}O_{184}]^{32-}$	89.7	90.3	90.3	89.7	
$Be_8[P_8W_{48}O_{184}]^{24-}$	87.6	90.6	90.5	91.3	
$Mg_8[P_8W_{48}O_{184}]^{24-}$	90.2	89.8	89.8	90.2	
$Ca_8[P_8W_{48}O_{184}]^{24-}$	90.0	90.0	90.0	90.0	

Table S26. Collection of calculated inner diameter dimensions for $K_n\{P_8W_{48}\}$ structures, with a set of empirical angles for comparison.

Formula	Inner Ring Diameter 1 (nm)	Inner Ring Diameter 2 (nm)	Mean Inner Ring Diameter (nm)	Reference
$[P_8W_{48}O_{184}]^{40-}$	0.977	1.017	0.997	*Appl. Organomet. Chem.* 2020, **34**, 5702
$Li_8[P_8W_{48}O_{184}]^{32-}$	1.061	1.013	1.037	
$Na_8[P_8W_{48}O_{184}]^{32-}$	1.0205	1.0495	1.035	
$K_8[P_8W_{48}O_{184}]^{32-}$	1.013	1.023	1.018	
$Rb_8[P_8W_{48}O_{184}]^{32-}$	1.017	1.028	1.0225	
$Be_8[P_8W_{48}O_{184}]^{24-}$	1.0485	1.061	1.055	
$Mg_8[P_8W_{48}O_{184}]^{24-}$	1.023	1.060	1.042	
$Ca_8[P_8W_{48}O_{184}]^{24-}$	1.031	1.029	1.030	

Table S27. Collection of calculated outer diameter dimensions for $K_n\{P_8W_{48}\}$ structures, with a set of empirical angles for comparison.

Formula	Outer Ring Diameter 1 (nm)	Outer Ring Diameter 2 (nm)	Mean Outer Ring Diameter (nm)	Reference
$[P_8W_{48}O_{184}]^{40-}$	1.644	1.646	1.645	*Appl. Organomet. Chem.* 2020, **34**, 5702
$Li_8[P_8W_{48}O_{184}]^{32-}$	1.680	1.673	1.6765	
$Na_8[P_8W_{48}O_{184}]^{32-}$	1.696	1.692	1.694	
$K_8[P_8W_{48}O_{184}]^{32-}$	1.704	1.704	1.704	
$Rb_8[P_8W_{48}O_{184}]^{32-}$	1.705	1.705	1.705	
$Be_8[P_8W_{48}O_{184}]^{24-}$	1.618	1.602	1.610	
$Mg_8[P_8W_{48}O_{184}]^{24-}$	1.659	1.659	1.659	
$Ca_8[P_8W_{48}O_{184}]^{24-}$	1.682	1.681	1.6815	

References

1. Vilà-Nadal, L. *et al.* Towards polyoxometalate-cluster-based nano-electronics. *Chem. - A Eur. J.* **19**, 16502–16511 (2013).

2. Cameron, J. M., Gao, J., Vilà-Nadal, L., Long, D. L. & Cronin, L. Formation, self-assembly and transformation of a transient selenotungstate building block into clusters, chains and macrocycles. *Chem. Commun.* **50**, 2155–2157 (2014).

3. Vilà-Nadal, L. *et al.* Polyoxometalate {W18O56XO6} Clusters with Embedded Redox-Active Main-Group Templates as Localized Inner-Cluster Radicals. *Angew. Chemie* **125**, 9877–9881 (2013).

4. Zhang, F. Q. *et al.* On the origin of the relative stability of wells-dawson isomers: A DFT study of α-, β-, γ-, α*-, β*-, and γ*-[(PO4)2W18O54] 6-anions. *Inorg. Chem.* **50**, 4967–4977 (2011).

5. Boyd, T. *et al.* POMzites: A Family of Zeolitic Polyoxometalate Frameworks from a Minimal Building Block Library. *J. Am. Chem. Soc.* **139**, 5930–5938 (2017).

6. Long, D. L., Abbas, H., Kögerler, P. & Cronin, L. Confined electron-transfer reactions within a molecular metal oxide 'Trojan Horse'. *Angew. Chemie - Int. Ed.* **44**, 3415–3419 (2005).

7. Long, D. L. *et al.* A redox-triggered structural rearrangement in an iodate-templated polyoxotungstate cluster cage. *Chem. Commun.* **49**, 9731–9733 (2013).

8. Ozawa, Y. & Sasaki, Y. Synthesis and Crystal Structure of [(CH3)4N]6[H3BiW18O60]. *Chem. Lett.* **16**, 923–926 (1987).
